# α-Synuclein vaccination modulates regulatory T cell activation and microglia in the absence of brain pathology

**DOI:** 10.1186/s12974-016-0532-8

**Published:** 2016-04-07

**Authors:** Josefine R. Christiansen, Mads N. Olesen, Daniel E. Otzen, Marina Romero-Ramos, Vanesa Sanchez-Guajardo

**Affiliations:** Neuroimmunology of Degenerative Diseases group, Department of Biomedicine, HEALTH, Aarhus University, Aarhus, Denmark; CNS Disease Modeling group, Department of Biomedicine, HEALTH , Aarhus University, Aarhus, Denmark; AU Ideas Pilot Center NEURODIN, Department of Biomedicine, HEALTH, Aarhus University, Aarhus, Denmark; Interdisciplinary Nanoscience Center - iNANO, Department of Molecular Biology and Genetics, Aarhus University, Aarhus, Denmark

**Keywords:** Dopamine receptor D2 (DR-D2), Dopamine receptor D3 (DR-D3), Parkinson’s disease, Foxp3, Neuroinflammation, Immunotherapy, Stat5, CCR6, CD103

## Abstract

**Background:**

Passive and active immunization with α-synuclein has been shown to be neuroprotective in animal models of Parkinson’s disease. We have previously shown that vaccination with α-synuclein, long before α-synuclein-induced brain pathology, prevents striatal degeneration by inducing regulatory T cell infiltration in parenchyma and antibody deposition on α-synuclein overexpressing neurons. However, the effect of peripheral α-synuclein on the immune system is unknown, as are the mechanistic changes induced in the CD4 T cell population during successful neuroprotective animal studies. We have studied the changes induced by vaccination with α-synuclein in the CD4 T cell pool and its impact on brain microglia to understand the immune mechanisms behind successful vaccination strategies in Parkinson’s disease animal models.

**Methods:**

Mice were immunized with WT or nitrated α-synuclein at a dose equivalent to the one used in our previous successful vaccination strategy and at a higher dose to determine potential dose-dependent effects. Animals were re-vaccinated 4 weeks after and sacrificed 5 days later. These studies were conducted in naive animals in the absence of human α-synuclein expression.

**Results:**

The CD4 T cell response was modulated by α-synuclein in a dose-dependent manner, in particular the regulatory T cell population. Low-dose α-synuclein induced expansion of naive (Foxp3 + CCR6-CD127lo/neg) and dopamine receptor type D3+ regulatory T cells, as well as an increase in Stat5 protein levels. On the other hand, high dose promoted activation of regulatory T cells (Foxp3CCR6 + CD127lo/neg), which were dopamine receptor D2+D3-, and induced up-regulation of Stat5 and production of anti-α-synuclein antibodies. These effects were specific to the variant of α-synuclein used as the pathology-associated nitrated form induced distinct effects at both doses. The changes observed in the periphery after vaccination with low-dose α-synuclein correlated with an increase in CD154+, CD103+, and CD54+ microglia and the reduction of CD200R+ microglia. This resulted in the induction of a polarized tolerogenic microglia population that was CD200R-CD54CD103CD172a+ (82 % of total microglia).

**Conclusions:**

We have shown for the first time the mechanisms behind α-synuclein vaccination and, importantly, how we can modulate microglia’s phenotype by regulating the CD4 T cell pool, thus shedding invaluable light on the design of neuroimmunoregulatory therapies for Parkinson’s disease.

**Electronic supplementary material:**

The online version of this article (doi:10.1186/s12974-016-0532-8) contains supplementary material, which is available to authorized users.

## Background

Parkinson’s disease (PD) is characterized by the loss of dopaminergic neurons in substantia nigra and the presence in surviving neurons of pathological α-synuclein (α-syn) aggregates termed Lewy bodies. Great effort has been put into understanding the role of α-syn in PD etiology and how to avert its detrimental effects. Several strategies have been designed to prevent neuronal α-syn accumulation or enhance clearance of α-syn aggregates in PD-like animal models. Some of these strategies have been designed to harness the adaptive immune system, either through generation of α-syn-specific antibodies to clear the α-syn deposits [1, 2] or via T cells in order to tip the adaptive immune response into a Foxp3+ regulatory T (Treg) cell phenotype [3, 4]. However, the actual effect that α-syn vaccination has on the peripheral adaptive immune system has not yet been investigated.

One of the characteristics of PD is the neuronal loss in substantia nigra and the consequent overt decrease in dopamine release. Interestingly, an overlooked aspect of the disease is the effect this lack of dopamine has on the immune system, as T cells express dopamine receptors (DRs) and the dopamine transporter [5–10]. Out of the five DRs, the type expressed by CD4 T cells appears to depend on the type of effector cell they differentiate into (i.e., Th1 vs. Th2) (reviewed in [11, 12]). Dopamine signaling influences the type of effector CD4 T cell generated, and its effect is dependent on whether dopamine is present when the T cell encounters its cognate antigen for the first time or as an effector/memory cell (reviewed in [13]). The concentration of dopamine in serum is estimated to be 10 pg/ml [5], but this is increased locally when T cells encounter dendritic cells and/or Treg cells, as both of these cell types produce dopamine [14–16]. Dopamine is able to oxidize α-syn and induce generation of toxic oligomeric species (for a review see [17]). At the same time, α-syn is present in serum and +cerebrospinal fluid (CSF), although the change in α-syn levels during PD is still controversial. Data exist showing α-syn increase [18] or decrease [19] in serum; as regards CSF, there is an initial consensus of its decrease in synucleinopathy patients [19]. However, there is an agreement that α-syn can be released from cells in its natural unfolded status or abnormally oligomerized [20–22]. Furthermore, anti-α-syn antibodies are found in serum, suggesting that α-syn is eliciting a sterile immune response in the peripheral immune system [23–25]. Sterile immune responses have been observed in Alzheimer’s disease and relate to early, non-TCR-mediated responses that result in inflammation in the absence of a pathogen. Thus, discerning how α-syn affects DR expression on T cells is of vital interest if we want to develop effective immunoregulatory therapies for PD, as T cells may not be responding the same way as in healthy conditions, and/or they may be altered by the dopamine replacement drugs currently used for PD treatment such as l-dopa. There is, in fact, accumulating evidence that the peripheral immune system in PD patients is affected [26–34], that α-syn is expressed in T cells [35], and that dopamine influences α-syn oligomerization and toxicity [36–39].

We have previously shown that vaccination with human α-syn (150 μg in 200–225g rats) 10 weeks before the onset of α-syn-induced brain pathology resulted in protection against neuronal pathology, which coincided with infiltration of Tregs into the brain parenchyma [3]. Our study contrasted with the adverse effect seen on 1-methyl-4-phenyl-1,2,3,6-tetrahydropyridine (MPTP)-induced neuronal pathology when the adaptive immune system was exposed to the pathology-related nitrated α-syn (Nα-syn) [40, 41]. These studies raised the question as to how α-syn variants change the CD4 T cell population in order for it to respond differently when challenged with neuronal overexpression/accumulation of α-syn and how this response is modified by pathology-associated modifications of α-syn. This was especially interesting since at the time of α-syn overexpression in our previous study (10 weeks post vaccination), the peripheral immune system was no longer active, suggesting that a recall adaptive immune response was mounted. Additionally, it remained unclear if vaccination per se had affected microglia or if the observed changes in microglia were mediated by the peripheral immune system upon α-syn accumulation. We elucidate herein the mechanisms behind our previous successful α-syn vaccination strategy to slow/avert α-syn-induced neuronal pathology, in particular, its effects on modulating the CD4 T cell pool and its impact on brain microglia, and compare them to the effects caused by the pathology-associated variant Nα-syn.

## Methods

### Animals and vaccination strategy

**Fig. 1:**
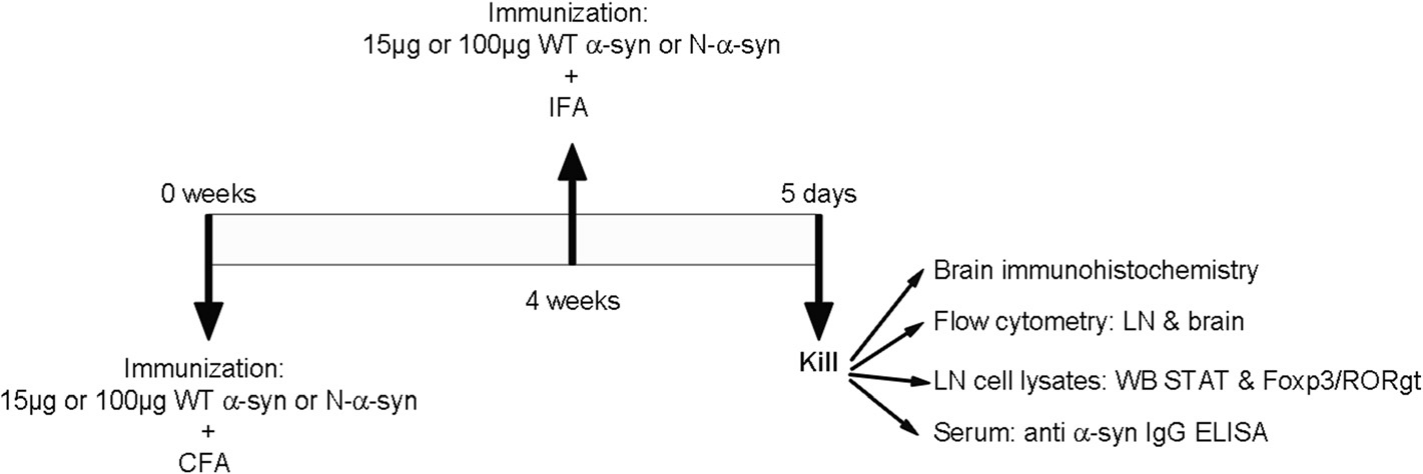
Schematic diagram of the experimental design. Foxp3-RFP mice were immunized with 15 or 100 μg of recombinant human α-syn in complete Freund’s adjuvant and re-immunized after 4 weeks with the same amount of protein in incomplete Freund’s adjuvant. Five days later, the animals were killed and the lymph nodes and brain dissected for analysis. Blood was obtained to determine antibody load in serum. A single lymph node cell suspension was made and either analyzed by flow cytometry or incubated for 6 h, after which the cells were lysed for analysis by Western blot. Microglia were isolated from brains and analyzed by flow cytometry. Alternatively, the animals were perfused at the time of death and brains processed for immunohistochemistry

A total of 58 10-week-old Foxp3-IRES-mRFP (FIR) mice (C57BL/6 background) [42] equally distributed between sexes were used divided into six groups of *n* = 8–10 animals per group. No statistical differences due to animal gender were observed throughout the project. The Foxp3-RFP mice were a kind gift from Prof. Antonio A. Freitas, Pasteur Institute, France. Our vaccination strategies consisted of recombinant human monomeric α-syn or its nitrated variant (Nα-syn), as this pathology-associated modification is known to exacerbate brain pathology. Mice were vaccinated with 15μg (the dose for mice equivalent to that used in our previous study in rats) or 100-μg protein (to assess dose effect) and 150 μl of complete Freund’s adjuvant subcutaneously on their back at the level of the base of the tail (total volume 300 μl). Four weeks after, the animals were again injected with the same amount of protein in incomplete Freund’s adjuvant. Five days after the second immunization, the animals were killed for analysis (Fig. 1). As control groups we also included the following: naive (not immunized, as a baseline to be able to determine if any immune processes took place) and adjuvant (only immunized with Freund’s adjuvant, to ascertain whether any differences were due to α-syn variants and not the adjuvant). The animals were distributed into eight independent experiments, with animals from different experimental groups included in each experiment; all groups contain animals of at least three independent experiments. Animal permits to perform the experiments have been approved by the Animal Inspectorate. All experimental animal work was conducted according to Danish regulations (Law no. 253, 08 03 2013, Executive order no. 88, 30 01 2013) in agreement with European Union directive (2010/63/EU) and under the guidance of the veterinarian of the Faculty of Health, Aarhus University.

### Protein preparations

Human recombinant α-syn was prepared as published before [43]. A fraction of the α-syn was nitrated in our laboratory following the protocol from Reynolds et al. [4]. Nitration was subsequently verified by Western blot (≥98 % of total α-syn) and mass spectrometry (kindly performed by Dr. Steen Vang Petersen, Department of Biomedicine, Aarhus University). All protein solutions were diluted at 1 μg/μl in isotonic NaCl at the time of injection to obtain a volume of 150 μl/mouse of protein solution.

### Lymph node cell preparation

At the time of death, the inguinal, axillar, and brachial lymph nodes were pooled per animal and single cell suspensions were made in complete medium (RPMI-glutamax with 10 % fetal calf serum (FCS), HEPES and penicillin/streptomycin, all from GIBCO). Cells were filtered through a 100-μm mesh and washed in 10 ml medium. After centrifugation (400g for 10 min), the cells were resuspended in 1 ml complete medium. An aliquot was taken for cell counting, and 100 μl were taken for flow cytometry analysis. The remaining cells were plated on round bottom plates and let to rest in an incubator for 6 h, after which the medium was recovered, and cells were lysed in complete lysis buffer (one cOmplete Mini protease inhibitor tablet, Roche Diagnostics and two phosSTOP tablets, Roche Diagnostics, per 10 ml lysis buffer: 10 mM Tris-base, 150 mM NaCl, 0.5 mM EDTA, 1 % IGEPAL CA-630 in deionized water). Both samples were frozen at −20 °C until further analysis.

### Microglia isolation

Brains were quickly dissected at the time of death and homogenized in 1.4 ml of HEPES-buffered saline (HBS) medium (GIBCO); 600 μl of Trypsin (50 mg/ml, Trypsin-EDTA, SIGMA) were added and the mix was put on a 37 °C water bath for 15 min. Two milliliters of HBS medium and 800 μl of FCS (20 %) were added before centrifugation (200*g* for 4 min). The pellet was resuspended in 5 ml HBS and carefully pipetted to obtain a single cell suspension. The sample was filtered (40 μm) before centrifugation, and the pellet was resuspended in 2.3 ml 75 % Percoll (GE Healthcare, Sweden). Five milliliters of 25 % percoll followed by 3 ml PBS were layered on top of the cell suspension, and the sample was centrifuged for 25 min at 800*g*. Microglia were recovered from the interphase between the 75 and 25 % gradient and washed with 15 ml PBS. After centrifugation, the pellet was resuspended in 100 μl PBS for fluorescence-activated cell sorting (FACS) analysis.

### Flow cytometry

All samples were plated on a 96-conical-well plate and centrifuged 4 min at 400*g*. The cells were then incubated for 10 min with 30 μl/well Fc block, centrifuged again, and resuspended in 30-μl antibody mix (Additional file 1: Table S1) for 10 min incubation in the dark. After washing, 30 μl/well of secondary antibody mix was added, and the cells were incubated for 10 min in the dark, washed twice, and resuspended in 200 μl buffer. All incubations were done on ice, and all the washes (100 μl, 300*g*), and antibody mix were done in PBS with 0.5 mM EDTA, penicillin/streptomycin, 2 % FCS without Ca^2+^ and Mg^2+^. Sample data was acquired in a FACS ARIA III (with four lasers) interphased to FlowJo software for analysis. All samples were gated first on live cells according to their FSC vs. SSC, doublets were removed by plotting FSC-H vs. FSC-A, and either 5000 CD3CD4Foxp3-RFP+ or 10,000 CD11b+-gated cells were acquired for analysis. The gates of positive CCR6, CD103, CD25, CD127, DR-D2, and DR-D3 cells were determined by the use of Fluorescence Minus One (FMO) for each of these antibodies.

### Immunohistochemistry

Three mice per group were killed with an overdose of pentobarbital and, upon respiratory arrest, perfused through the ascending aorta with ice-cold saline solution (without heparin) followed by 4 % paraformaldehyde. The brains were post-fixed in paraformaldehyde for 4 h and left for cryoprotection in a 25 % sucrose solution. The brains were then sliced into 40-μm-thick coronal sections and separated into four full brain series. Immunohistochemistry was performed on a full series for α-syn, and in one third of a series for CD11b, anti-mouse IgG, major histocompatibility complex (MHC) II, and CD4 (for antibody specification see Additional file 1: Table S1). Free-floating sections were quenched for 20 min in a solution of 3 % hydrogen peroxide and 10 % methanol. One hour of pre-incubation with 5 % appropriate normal serum was followed by overnight incubation at room temperature with the primary antibody in 2.5 % normal serum. Thereafter, the sections were incubated for 2 h with the appropriate biotinylated secondary antibody in 1 % normal serum. Sections were further incubated 1 h with avidin-biotin-peroxidase complex in PBS (ABC Elite, Vector Laboratories, Burlingame, CA). Sections were rinsed three times in potassium-phosphate buffer (KPBS) between each incubation period. All incubation solutions contained 0.25 % Triton X-100 in KPBS. Visualization was done using 3,3-diaminobenzidine (DAB) and 0.1 % of hydrogen peroxide for α-syn visualization and 0.01 % for the others. The sections were mounted on chrome-alum-coated glass slides and cover-slipped. Sections were analyzed by an observer blind to the identity of the samples on a Zeiss LSM710 microscope at ×1.25, ×10, and ×40; photographs were taken with VisioPharm software.

### SDS-PAGE and Western blot

Prior to SDS-PAGE and Western Blot analyses, the concentration of protein in the samples was determined using a bicinchoninic acid assay with a bovine serum albumin standard curve. One hundred microgram of protein was mixed with an SDS- and DTE-containing loading buffer prior to boiling and loading on an 8 % Bis-Tris gel (VE vertical electrophoresis system, Hoefer). After separation, proteins were blotted onto ethanol pre-activated polyvinylidene fluoride membrane (PVDF) membranes (GE Healthcare) for 1.5 h in an ethanol-containing buffer using the Hoefer system. Thereafter, membranes were blocked with 5 % skimmed milk in Tris-buffered saline with 0.05 % Tween-20 (TBS-T) for 1 h at room temperature and then incubated with a primary antibody (Additional file 1: Table S1) in 1 % skimmed milk TBS-T solution overnight at 4 °C. On the following day, the membranes were washed three times for 5 min with TBS-T and incubated with the appropriate horseradish peroxidase (HRP)-conjugated secondary antibody (Additional file 1: Table S1) in 1 % milk in TBS-tween for 2 h at room temperature. The blots were visualized by enhanced chemiluminiscence (Amersham ECL Western Blotting detection reagents, GE Healthcare) using a Fuji LAS-4000 ImageReader. Subsequently, blots were stripped for 30 min at 50 °C and incubated with a different primary antibody. The intensities of the protein bands were quantified using MultiGauge software and normalized to the level of β-actin in the sample. The Foxp3 antibody detected two bands at 50 kDa in some samples, which is believed to present a full-length isoform and an isoform lacking exon 2, respectively. Both Fopx3 variants have been shown to function as inhibitors of CD4 T cell activation [44], and thus when quantified, the intensities of both Foxp3 bands in a sample were added up.

### Anti-α-synuclein antibody titration by ELISA

Prior to perfusion, retro-orbital blood samples were taken and allowed to coagulate for 24–48 h at 4 °C and centrifuged for 10 min at 400*g*. Serum was isolated and stored at −20 °C until analysis. The serum titer of anti-α-syn antibodies was analyzed by indirect ELISA. MaxiSorp 96-well ELISA plates (Thermo Fisher Scientific) were coated overnight at 4 °C with 200 ng/well recombinant human α-syn (the same used to immunize the animals) in 100 mM carbonate/bicarbonate buffer, pH 9.6. After three washing steps using PBS with 0.05 % Tween-20 (PBS-T), wells were blocked for 2 h with 150 μL 0.2 % bovine serum albumin and 0.05 % Tween-20 in PBS (referred to as “blocking buffer”). Next, 100 μL of serum samples serially diluted in blocking buffer were added for overnight incubation at 4 °C. Additionally, monoclonal mouse anti-human α-syn IgG (Covance, clone 4B12) was used from 1:1000 to 1:128,000 to allow generation of a standard curve. All dilutions were run in duplicates. After rinsing in PBS-T, wells were incubated for 2 h at room temperature with 100 μL horseradish peroxidase-conjugated monoclonal rabbit anti-mouse IgG in blocking buffer. As negative controls, either standard antibody or secondary antibody was omitted during incubations. Following three washes in PBS-T, plates were allowed to develop for 20–25 min with 100 μL 1-Step Ultra TMB-ELISA Substrate (Thermo Fisher Scientific) and reaction stopped by addition of 100 μL ready-to-use sulfuric acid Stop Solution (Thermo Fisher Scientific). Absorbances at 450 nm were read on a VersaMax plate reader (Molecular Devices). A log decay curve was fitted within the linear range (1:2000–1:48,000). This fit and absorbance measured from serum diluted at 1:400 or 1:3200 were used to calculate serum anti-α-syn titer (μg/μL).

### Statistical analysis

Statistical comparison of data was performed using Prism 6 (GraphPad Software, Inc). A parametric, one-way ANOVA assuming no matching/pairing of data and equal SD was done for all studies. When significant, it was followed by multiple comparisons with a Tukey pos hoc analysis, or in the case of the distribution analysis (Figs. 5 and 6), a Fisher’s least significant difference (LSD) to determine significant changes between groups. Significance was accepted at the 95 % probability level.

## Results

We immunized naive Foxp3-RFP mice with human recombinant α-syn at two different doses: low, equivalent to the one used in our previous study, and high, to determine potential dose-dependent effects. As controls, we immunized additional groups of mice with adjuvant alone to determine its contribution to the response as it has been shown to be protective per se in PD animal models [45, 46] and the pathology-associated Nα-syn to show that the response is specific for wild type α-syn. Animals were re-immunized 4 weeks after the original vaccination and killed 5 days later to study the T cell response and changes in brain microglia (Fig. 1). A group of naive animals was included as an additional control group to determine the baseline of all immunological parameters, such as cell numbers, percentage and distribution of cell populations, and activation states. This allows the determination of any immunological change as compared to the homeostatic state, indicative of an immune response.

### α-Synuclein vaccination decreases the percentage of CD3+CD4- T cells and increases the number of CD3CD4Foxp3+ cells

**Fig. 2:**
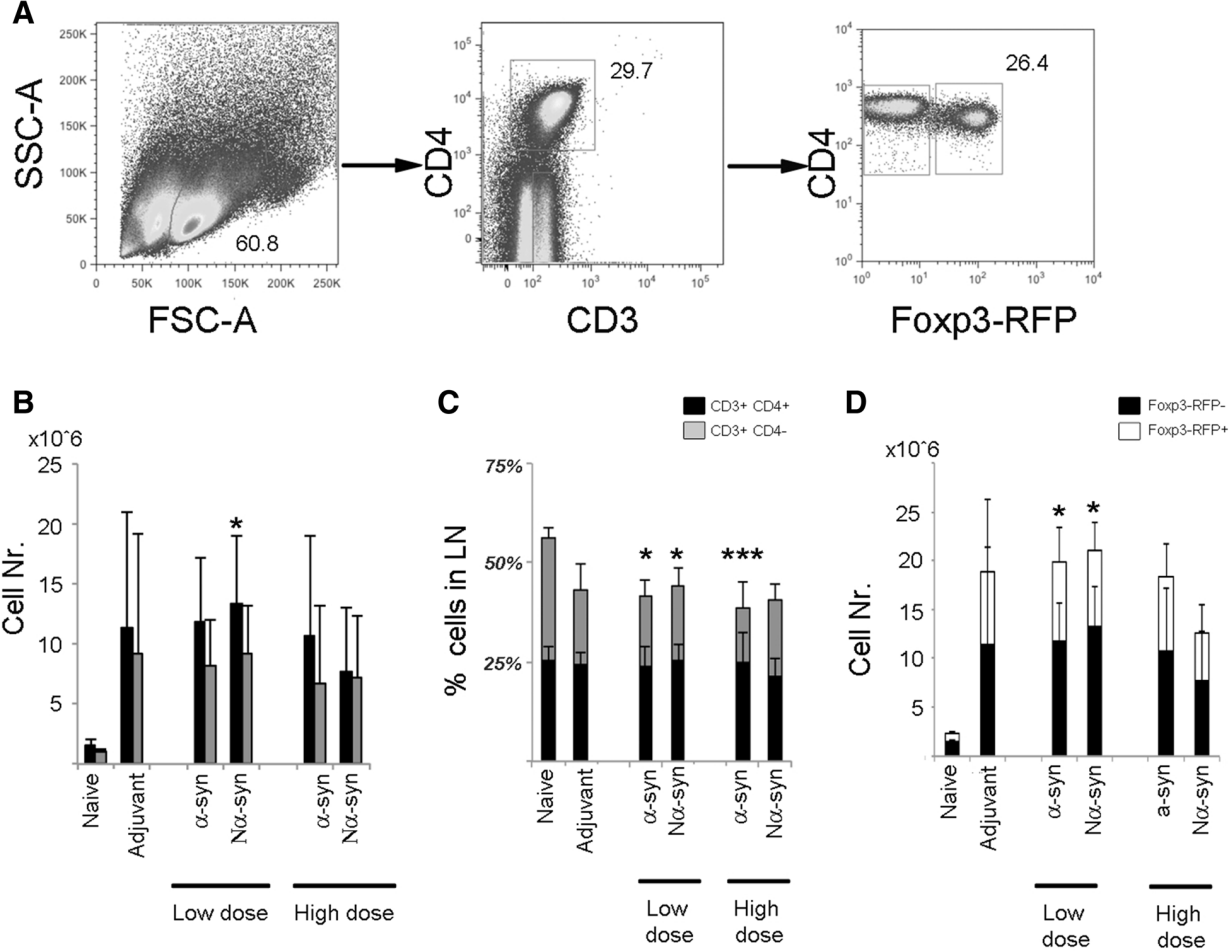
T cell numbers in lymph nodes. Lymph node cells were analyzed by flow cytometry. **a** Representative dot plots of gated live cells, thereafter gated for CD3+CD4+ and CD3+CD4-. The CD4+ fraction was further gated into Foxp3+ and Foxp3− cells. **b** Total number of CD3+CD4+ and CD3+CD4- cells. **c** Percentage of CD4+ and CD4− cell in lymph nodes. **d** Total CD3+CD4+Foxp3+ cells within the total CD3+CD4+ cell population. One-way ANOVA followed by Tukey HSD. *Asterisk* means different from naive. *p* < 0.05. All numbers are average + SD. *N* = 8–10, divided into three to five independent experiments

The percentages of live CD3+CD4+ and CD3+CD4- cells (assumed CD8+ lymphocytes), as well as the percentage of Foxp3+ cells within the CD4 T cell pool in lymph nodes, were assessed by flow cytometry (Fig. 2a). The number of T lymphocytes was generally increased to a highly variable degree upon vaccination, but only low-dose Nα-syn gave a significant increase in CD3CD4+ cells as compared to naive (Fig. 2b). However, vaccination with low or high dose of α-syn resulted in a significant reduction of the percentage of CD3+CD4- cells (Fig. 2c), indicating that the response was mainly of the CD4 T cell type. Furthermore, the number of CD3CD4Foxp3+ cells was significantly increased upon low-dose vaccination independently of the α-syn variant (Fig. 2d).

### α-Synuclein affects the naive/activated/memory distribution of Foxp3+ T cells

**Fig. 3:**
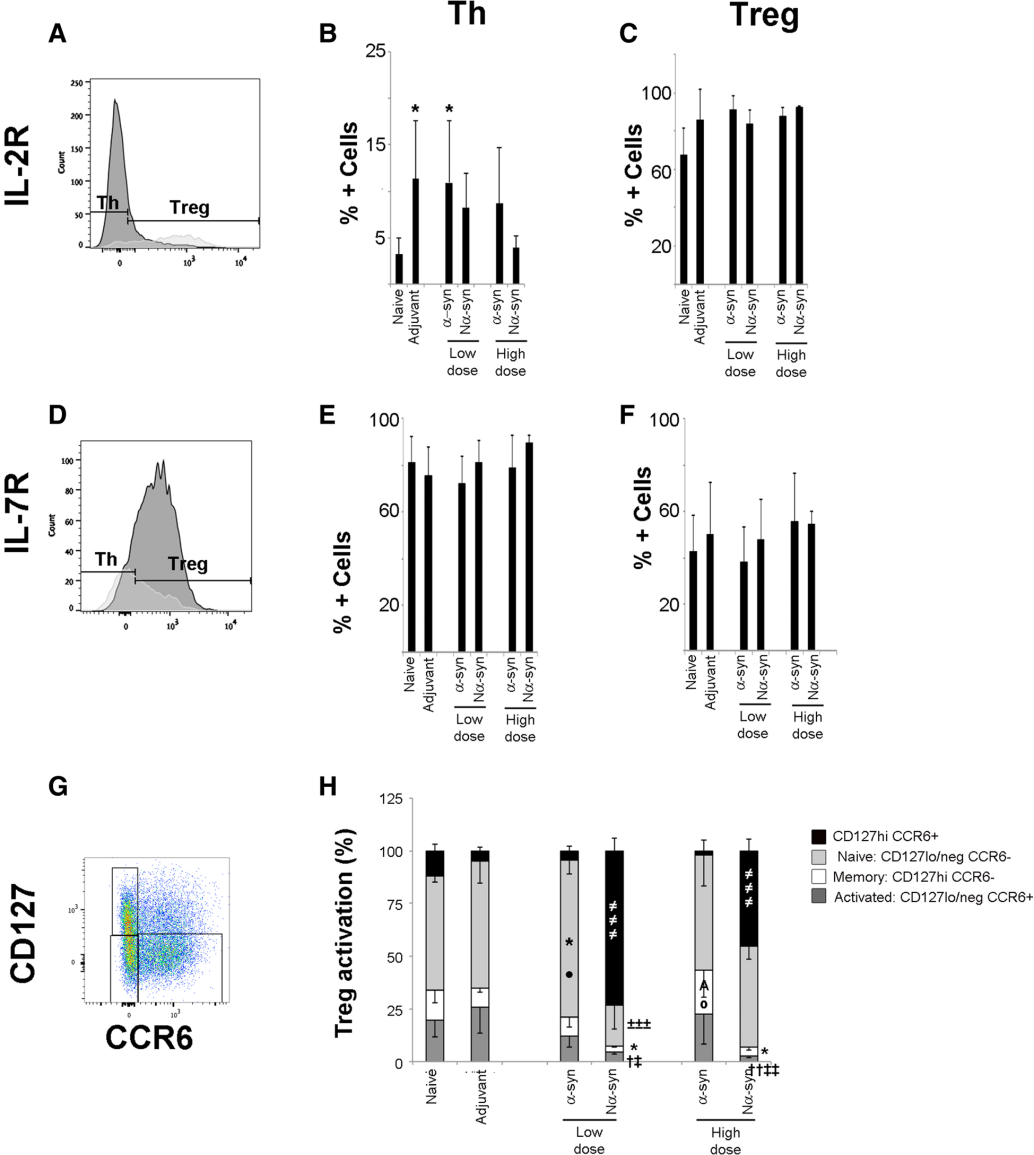
IL-2Ra (CD25) and IL-7Ra (CD127) expression on CD3+CD4+ lymph node cells. Cells were gated for CD3+CD4+Foxp3- (i.e., Th) and CD3+CD4+Foxp3+ (i.e., Treg) as in Fig. 2. Representative histograms showing the level of expression of IL-2Ra (**a**) and IL-7Ra (**d**) in Th (*dark gray*) and Treg (*light gray*) T cell populations. *Bar graphs* showing the average percentage + SD of cells expressing CD25 (**b**, **c**) or CD127 (**e**, **f**). **b**, **e** CD3+CD4+Foxp3- cells. **c**, **f** CD3+CD4+Foxp3+ cells. **g** Representative *dot plot* of CD3+CD4+Foxp3+ cells expressing CD127 and CCR6. **h** *Bar graphs* showing the distribution of CD3+CD4+Foxp3+ cells (average percentage + SD) according to their expression of CD127 and CCR6. One-way ANOVA followed by Tukey HSD. *Asterisk* means different from naive; *A* means different from adjuvant; *dagger symbol* means different from naive and adjuvant; *closed circle* means different from high-dose α-syn and Nα-syn; *open circle* means different from the other α-syn variant independent of dose; *not equal to symbol* means different from all; *double dagger symbol* means different from the other α-syn variant at a different dose; *plus-minus sign* means different from the same α-syn variant at different dose. *p* < 0.05. *N* = 8–10, divided into three to five independent experiments

To examine the effect of α-syn on T cell activation and survival, the number of cells expressing the IL-2Ra (CD25) within the CD3+CD4+ T cell pool was estimated by flow cytometry (Fig. 3a). The percentages of activated CD3+CD4+ T cells 5 days after re-vaccination were significantly different from naive when the animals were vaccinated with low-dose α-syn or adjuvant alone (Fig. 3b). Nα-syn, on the other hand, showed a tendency to abolish this CD25 up-regulation (induce activation), especially at high dose. Vaccination, however, had no effect on the survival capacity of Foxp3+ T cells (Fig. 3c).

We also measured IL-7Ra (CD127, Fig. 3d), as a marker of T cell effector/memory survival (Fig. 3e) and Treg activation (Fig. 3f), but vaccination had no effect on these populations. However, when the distribution between naive (CD127lo/negCCR6-), activated (CD127hiCCR6-), and memory (CD127lo/negCCR6+) Foxp3+ T cells was analyzed (Fig. 3g), a significant increase in the percentage of naive Foxp3+ T cells was observed in animals vaccinated with low-dose α-syn with respect to naive and both α-syn variants at high dose (Fig. 3h). High-dose α-syn, on the other hand, expanded the memory fraction of Treg as compared to adjuvant and Nα-syn independent of the dose. Nα-syn significantly increased the fraction of the double-positive population irrespective of the dose when compared to all other groups. Importantly, these changes were significantly different to adjuvant (A, †, ≠ in Fig. 3), thus indicating that it is a direct response to the α-syn variant and not due to the administration of adjuvant.

To our knowledge, the presence of both markers has not been associated with any specific activation state or function of the Treg population, and thus, this could indicate that Nα-syn has switched off/inactivated the Treg compartment.

### α-Synuclein variants tend to modify the RORγt–Foxp3 balance and alter Stat protein expression

**Fig. 4:**
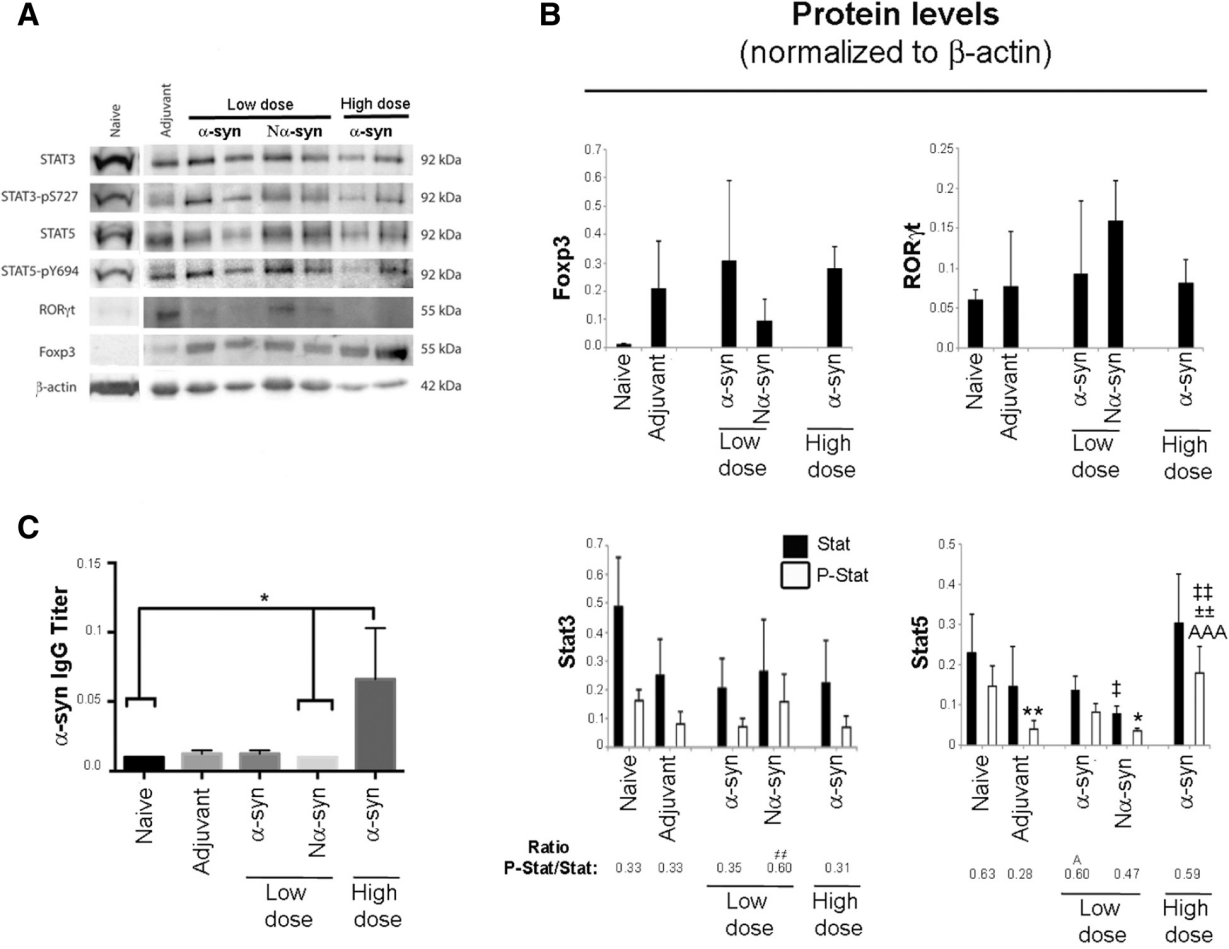
T cell differentiation. **a** Lymph nodes cells were incubated in vitro for 6 h without activation and thereafter lysed for analysis by western blot as described in the “Methods” section. Representative blot for each protein studied. Band intensity was quantified and normalized to the intensity of β-actin. **b** *Bar graphs* showing the average relative value + SD of normalized levels of protein in lymph node cell lysates (*n* = 3–5 samples/group). **c** *Bar graphs* showing the average + SD titer of anti-α-syn antibodies in serum (μg/μL). All samples were done in duplicates (*n* = 3–5/group). One-way ANOVA followed by Tukey HSD. *Asterisk* means different from naive; *A* means different from adjuvant; *not equal to symbol* means different from all; *double dagger symbol* means different from the other α-syn variant at a different dose; *plus-minus sign* means different from the same α-syn variant at different dose. *p* < 0.05

To determine whether α-syn modified the induction of Th17 cells (autoimmune inflammation) and Tregs (tolerance), we measured the levels of their respective canonical transcription factors RORγt and Foxp3. Additionally, we looked at the levels of Stat3 and Stat5 and their phosphorylation states because TGFβ/IL-6 and IL-2 signal through these molecules to induce, respectively, the Th17 or Treg phenotype (Fig 4a). All immunizations tend to increase Foxp3 levels compared to naive, but only low-dose α-syn showed a clear trend to its up-regulation as compared to naive (Fisher’s LSD, *p* = 0.0419), so the effect was probably due to the adjuvant (Fig 4b). Vaccination with Nα-syn was the only strategy that showed a trend to induce RORγt protein as compare to naive (*p* = 0.08, Fisher’s LSD). No significant changes in the protein level of Stat3 were observed among the groups, despite clear trends towards modulation of its phosphorylation (one-way ANOVA, *p* = 0.047); indeed, the ratio of phosphorylated Stat3 was significantly increased by Nα-syn as compared to all other groups (Fig 4b). Conversely, total Stat5 was significantly modulated depending on vaccination strategy (one-way ANOVA, *p* = 0.0002), although only the α-syn high-dose group showed significance as compared to low-dose Nα-syn. However, the level of Stat5 phosphorylation of the α-syn high-dose group was significantly increased as compared to low-dose α-syn for both variants and to adjuvant (Fig 4b), indicating that the changes in phosphorylation are due to the dose of α-syn and not the effect of the adjuvant. Indeed, the level of Stat5 phosphorylation of Nα-syn and adjuvant were significantly lower than the naive group. Together, these data suggest that vaccination with α-syn increases Stat5 and its phosphorylation in a dose-dependent manner.

### α-Synuclein vaccination only generates antibodies against α-synuclein at high dose

Serum was tested for anti-α-syn antibodies by ELISA (Fig. 4c). Only vaccination with high-dose α-syn showed a significantly higher serum titer of antibodies specific for α-syn as compared to naive and low-dose Nα-syn. This indicates that the immune response elicited by vaccination with a low dose of antigen did not involve a humoral response and thus, most likely, did not result in activation of the B cell pool by T cells, suggesting a suboptimal TCR activation. At high dose, however, we achieved an antigen-specific immune response against α-syn.

### Dopamine receptors on CD4 T cells are affected by dose and variant of α-synuclein

**Fig. 5:**
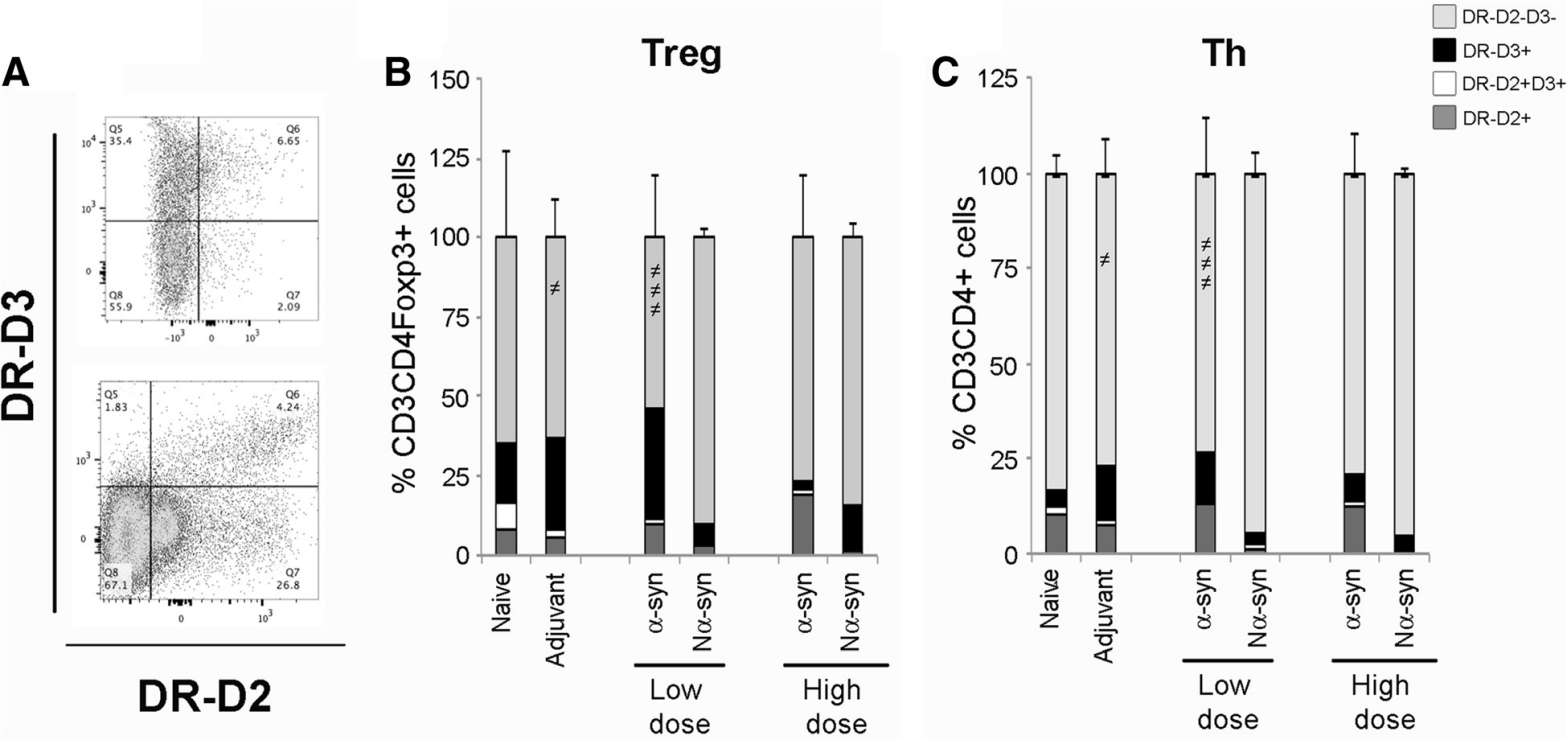
Dopamine receptor D2 and D3 expression on CD3+CD4+ cells. Th and Treg were gated as described, and the percentage of cells positive for dopamine receptor DR-D2 and DR-D3 were determined. **a** Representative *dot plots* for DR-D2 and DR-D3 co-expression. The relative percentages of DR-D2+, DR-D3+, double-positive (DR-D2+D3+), and double-negative (DR-D2-D3-) cells within the Treg (**b**) and Th (**c**) populations are shown as average + SD. One-way ANOVA followed by Fisher’s LSD. *not equal to symbol* means different from all. *p* < 0.05. *n* = 8–10, divided into three to five independent experiments

Dopamine is known to be a contributing factor in lymphocyte regulation, and at the same time, dopamine can form complexes with α-syn and increase its oligomerization [37, 47–49]. We have looked at two DRs known to have opposite effects on CD4 T cells (Fig. 5a): DR-D2, which is associated with T cell regulatory functions, and DR-D3, which is thought to induce pro-inflammatory responses and has been implicated in the detrimental T cell response in the MPTP model of PD [50, 51].

In general, vaccination induced a variable regulation of the DRs. However, some effects were observed: low-dose α-syn vaccination significantly decreased the DR double-negative fraction of the Tregs cells as compared to all other strategies, something also observed to a lesser extent in the adjuvant group (Fig. 5b). This correlated with a non-significant increase in DR-D3 expression. Increasing the dose of α-syn did not show a significant change in the percentage of DR negative cells but a tendency to increase DR-D2 at the expense of DR-D3. Nα-syn had a tendency to decrease the number of Tregs expressing DR-D2 and to increase the DR double-negative percentage independently of dose (one-way ANOVA, *p* = 0.07, Fisher’s LSD different to other groups).

When looking at effector CD4 T cells (Fig. 5c), we saw a similar decrease in the DR double-negative fraction as a result of adjuvant and low-dose α-syn vaccination, but in these populations, the tendency was to increase both DR-D2 and DR-D3, while eliminating the double-positive fraction. As for Tregs, Nα-syn abolished the expression of DR-D2.

### α-Synuclein modulates the homing (CCR6) and tolerance (CD103) capacity of CD4 T cells

**Fig. 6:**
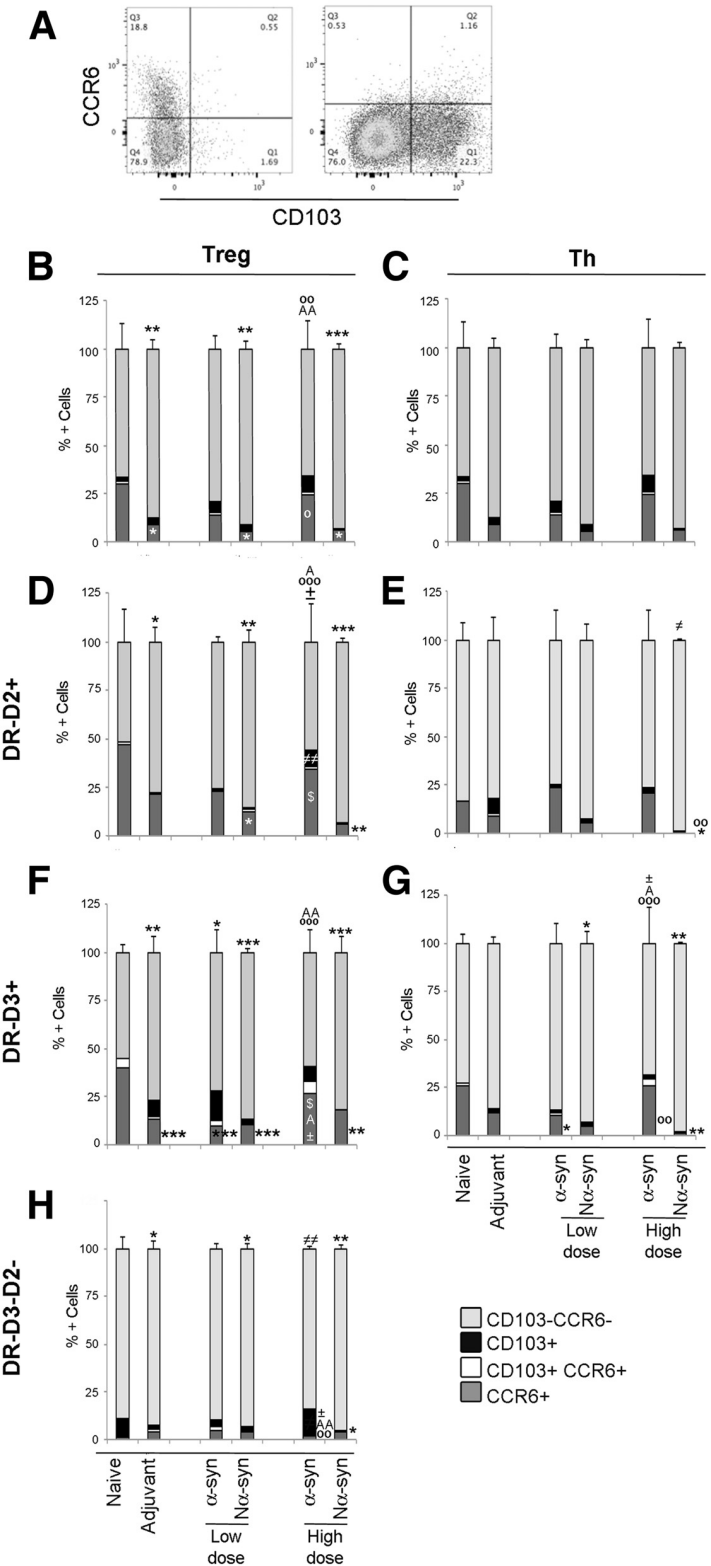
Expression of CCR6 and CD103. Cells were gated as before for CD3+CD4+Foxp3- (Th) and CD3+CD4+Foxp3+ (Treg) and thereafter for dopamine receptor expression: D2 (DR-D2+), D3 (DR-D3+) or double-negative (DR-D3-D2-). The expression of CCR6 and CD103 was determined in each of these populations. **a** Representative *dot plot* showing CCR6 and CD103 co-expression in Treg cells un-gated for DR. **b**–**g** *Bar graphs* representing the average percentage + SD of cells expressing CCR6 and CD103 in the total Treg (**b**) and Th (**c**) cell populations and in cells gated for DR-D2 (**d**, **e**) and DR-D3 (**f**, **g**). **h** *Bar graph* showing the average percentage + SD of CCR6+ and CD103+ cells in Treg cells negative for DR expression. One-way ANOVA followed by Fisher’s LSD. *Asterisk* means different from naive; *A* means different from adjuvant; *open circle* means different from the other α-syn variant independent of dose; *not equal to symbol* means different from all; *plus-minus sign* means different from the same α-syn variant at different dose; *dollar sign* means different from the other α-syn variant at the same dose. *p* < 0.05. *N* = 8–10, divided into three to five independent experiments

To determine whether α-syn vaccination had modified the capability of T cells to migrate to the brain, we measured the expression of CCR6, a chemokine receptor implicated in T cell brain homing in the animal model of multiple sclerosis (EAE) [52]. We also looked at the expression of CD103, a lectin involved in cell-cell induced tolerance and T cell extravasation (Fig. 6a) [53, 54]. The fraction of cells within the Treg (Fig. 6b) and Th (Fig. 6c) pools positive for any of these markers was variable but always below 40 %.

Treg expression of these markers was affected in a dose- and variant-dependent manner. Significant changes were observed in the number of double-negative cells (CD103-CCR6-), which were increased by adjuvant and Nα-syn vaccinations independently of dose with a concomitant significant reduction in CCR6+ cells. In contrast, high dose increased the CCR6+ fraction with respect to adjuvant and Nα-syn independently of dose, leading to a significant decrease in the double-negative fraction (Fig. 6b).

Interestingly, we observed that CD103 and CCR6 expression was mainly observed in DR-D2+ and DR-D3+ cells (though there is no direct correlation with DR expression), as only Th cells positive for these DRs expressed these markers (Fig. 6e, g), and the fraction of DR-D2-D3- Tregs expressing these markers was below 13 % (Fig. 6h). DR-D2 expression was mainly associated with expression of CCR6 (Fig. 6d, e), and only Tregs showed modulation of its expression upon vaccination. High-dose α-syn significantly induced CD103 on Tregs as compared to all other groups (Fig. 6d), while Nα-syn independently of dose significantly reduced CCR6 expression. DR-D3+ Tregs (Fig. 6f), on the other hand, expressed CCR6 but vaccination induced its down-regulation and promoted CD103 expression. This was mainly independent of vaccination strategy and thus most likely an effect of the adjuvant, except at high-dose α-syn, which induced CD103 without reducing CCR6 in an adjuvant-independent manner.

### Vaccination induces microgliosis specifically in substantia nigra

**Fig. 7:**
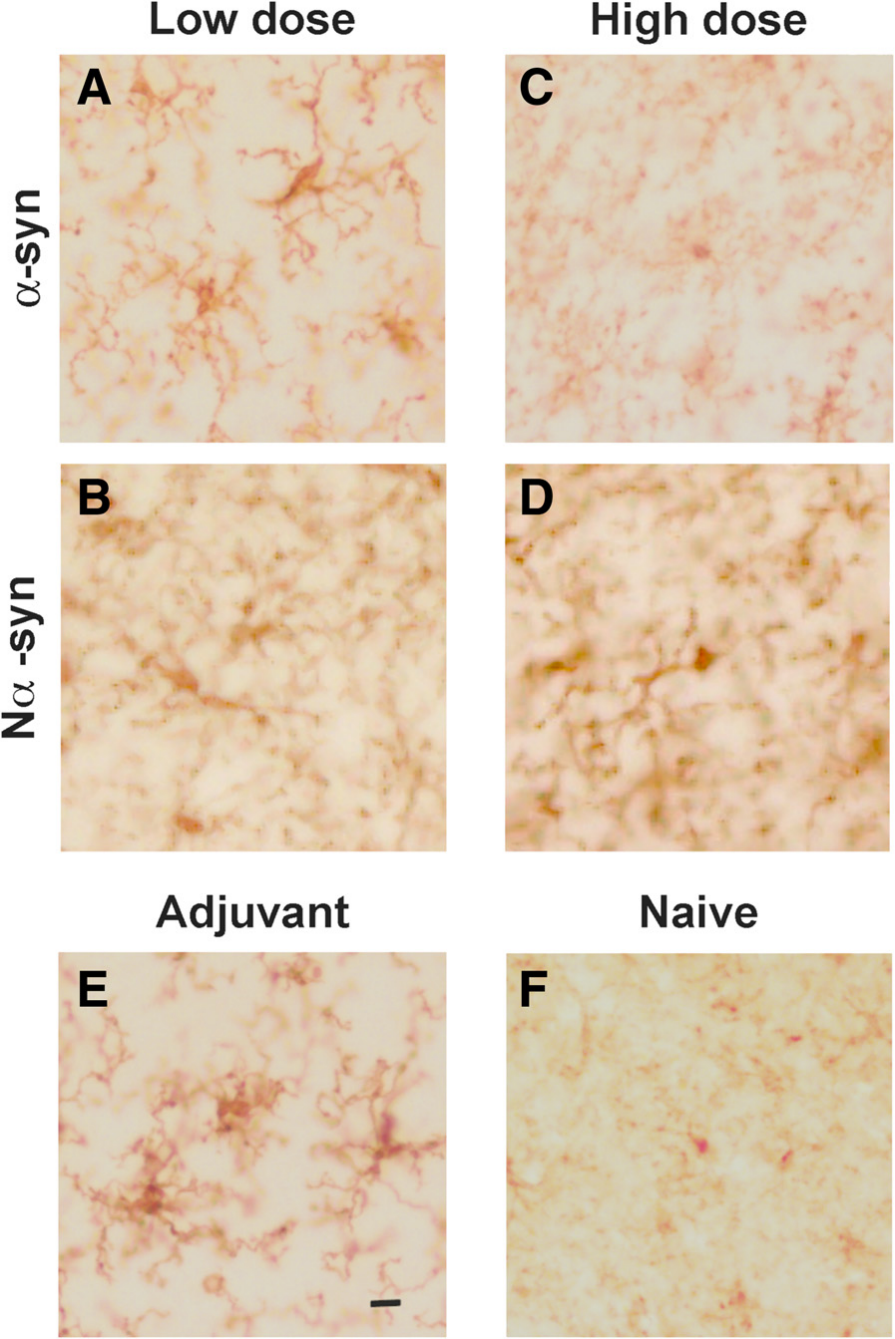
CD11b immunopositive microglia in substantia nigra. A series of coronal brain sections were immunostained with anti-CD11b antibody to assess changes in microglia cell number or morphology. *Photos* show representative substantia nigra images from animals having received low-dose protein (**a**, **b**), high-dose protein (**c**, **d**), and adjuvant (**e**) and from a naive mouse (**f**). *Scale bar* in **e** applies to all, 10 μm (*n* = 3, one experiment)

To determine if α-syn vaccination resulted in activation of brain microglia, a series of coronal sections throughout the brains were stained for CD11b as a marker of microglia. An observer, blind to the sample’s identity, analyzed the sections to assess any change in the morphological profile of CD11b+ microglia. With the exception of substantia nigra (Fig. 7), selected regions of the brain (cortex, hippocampus, and striatum) showed no overt difference as compared to naive animals (Additional file 2: Figure S1). In substantia nigra, however, animals that received adjuvant (Fig. 7e) presented microglia with enlarged soma, as well as elongation and hyper-ramification of their processes as compared to naive animals. These morphological differences were also observed in α-syn low dose (Fig. 7a), but not in α-syn high dose (Fig. 7c), suggesting that although microgliosis may be an effect of the adjuvant, the dose of the antigen also plays a role in how brain microglia reacts. Indeed, Nα-syn also induced changes in microglia morphology (Fig. 7b, d), but these changes were clearly different from those induced by adjuvant and α-syn as the soma was constricted, with barely any cytoplasm around the nucleus, and the processes were not branched. We also analyzed the expression of MHC II in adjacent sections, but no clear difference were observed among groups, with only occasional ramified MHC II+ microglia-like cells found in parenchyma in all groups (Additional file 3: Figure S2A). Indeed, most of the MHC II+ staining was associated to blood vessels in all groups (Additional file 3: Figure S2B).

We did observe sporadic microglia-like cells positive for CD4 (Additional file 3: Figure S2C&D) and blood vessel positive staining (Additional file 3: Figure S2E). However, no infiltrated CD4+ T cell (small, round, and un-ramified) were observed in brain parenchyma, suggesting that microglia activation was not induced by direct T cell-microglia interactions at the time of the sacrifice. This is not surprising, as there is neither the antigen nor a pathological process ongoing in the brain and thus no cues to make T cells home to the brain. Furthermore, this supports our hypothesis that the homing of CD4 T cells into the brain previously reported by us in the rAAV-α-syn PD model is the direct consequence of α-syn-induced pathology in brain.

### α-Synuclein does not cross the blood-brain-barrier and deposit in the brain

An obvious question when considering the effect of α-syn vaccination on microglia is whether α-syn crossed the blood-brain-barrier and deposited in the brain; if so, the changes in microglia might then not be the result of acting on the peripheral immune system but rather a direct consequence of α-syn accumulation in the brain parenchyma. When representative brains sections were immunostained for human α-syn 5 days after the last immunization, we were not able to detect any positive staining for the human protein throughout the brain in any group, and they were indistinguishable from naive animals (Additional file 4: Figure S3). We also stained series of brain sections for anti-mouse IgG, but with the exception of sporadic staining in the hippocampus of the α-syn high dose, no apparent IgG deposition was observed in brain parenchyma, confirming that α-syn-specific antibodies were not generated, and those generated at α-syn high dose did not find their antigen in brain (Additional file 3: Figure S2F). Indeed, blood vessels in all groups stained positive for IgG (Additional file 3: Figure S2G), indicating that antibodies did not cross the brain-blood-barrier.

### α-Synuclein low dose induces the polarization of brain microglia into a specific phenotype

**Fig. 8:**
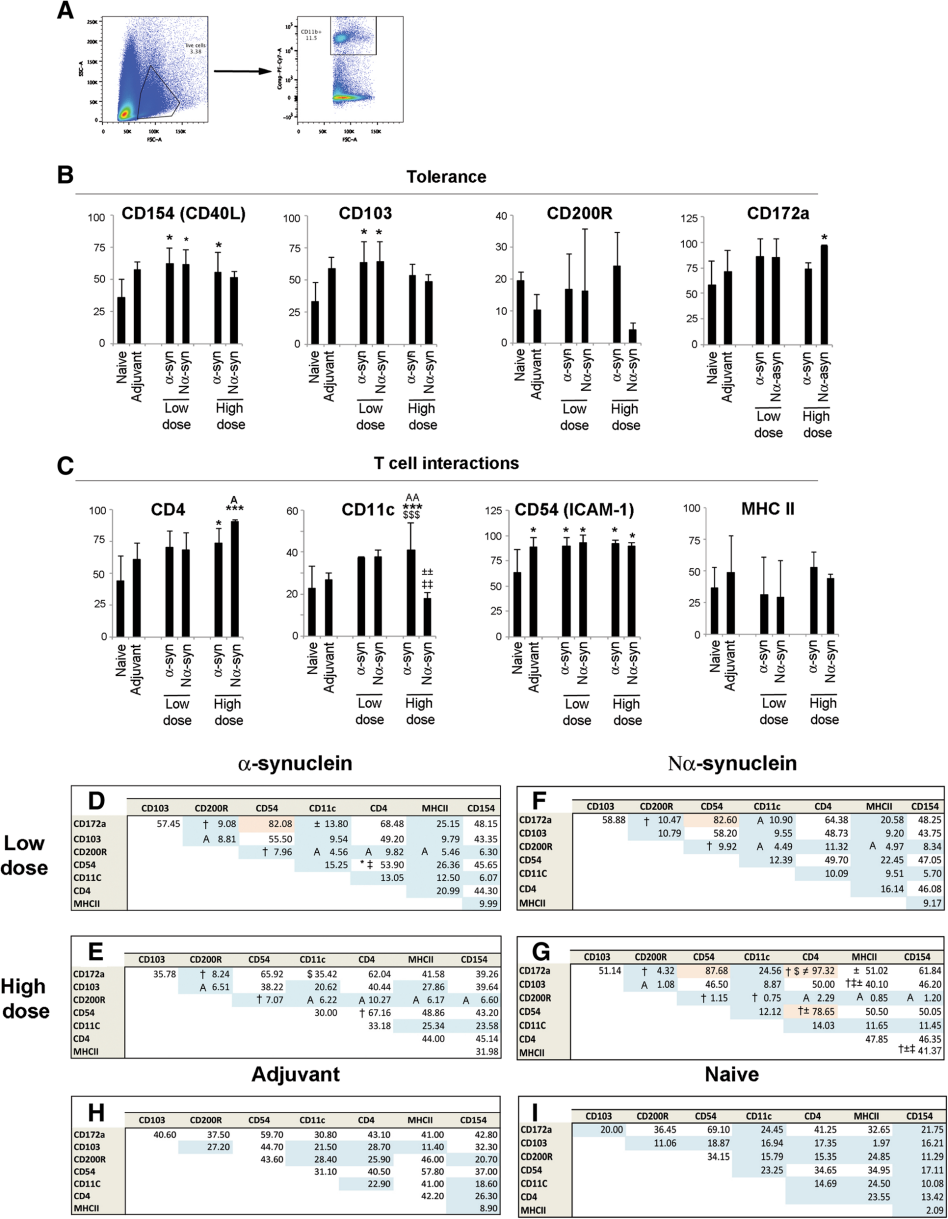
Percentage of microglia expressing diverse activation markers. **a** Representative *dot plots* showing the gating strategy for live CD11b+ cells. *Bar graphs* representing the average percentage + SD of microglia expressing activation markers related to tolerance (**b**) or adaptive immunity (**c**). *Tables* showing the average percentage of co-expression between pairs of activation markers at low dose (**d**, **f**) and high dose (**e**, **g**), as well as adjuvant (**h**) and naive (**i**). Markers co-expressed in less than 30 % of the population are in *blue* (considered to have an independent expression), and those co-expressed in 70 % or more are in *orange* (considered to have a correlated expression). One-way ANOVA followed by Tukey HSD. *Asterisk* means different from naive; *A* means different from adjuvant; *dagger symbol* means different from naive and adjuvant; *open circle* means different from the other α-syn variant independent of dose; *not equal to symbol* means different from all; *plus-minus sign* means different from the same α-syn variant at different dose; *dollar sign* means different from the other α-syn variant at the same dose; *double dagger symbol* means different from the other α-syn variant at a different dose. *p* < 0.05. *n* = 8–10, divided into three independent experiments

We were particularly interested in elucidating whether by altering the peripheral T cell pool we would modify the microglia phenotype. Total brain microglia were isolated from immunized mice (Fig. 8a) and stained with markers related to tolerance induction (Fig. 8b) or to interactions with the adaptive immune system (Fig. 8c) (see Additional file 1: Table S1 for function of each marker).

The percentage of microglia expressing the different markers varied depending on the dose and variant of α-syn. While CD154+ (CD40L) cell count was increased by α-syn independently of the dose, it only increased the percentage of CD103+ cells at low dose and CD4+ and CD11c+ cells at high dose (Fig. 8b, c), the latter being significantly higher than adjuvant. Nα-syn also showed a dose-dependent effect increasing the percentage of CD154+ and CD103+ cells at low dose (thus behaving as α-syn), but at high dose, it increased CD172a+ and CD4+ cells (significantly different from adjuvant), while decreasing CD11c+ percentage, showing variant specificity (Fig. 8b, c). All these effects were antigen-specific as adjuvant did not show any significant changes in these markers with respect to naive. The only change that seemed to be adjuvant-induced was the increase in CD54 (ICAM-1), which was similar across all vaccination strategies (Fig. 8c).

We analyzed the correlation between markers to determine if a specific microglia response was induced or if a plethora of distinct microglia were present (Fig. 8d–i). We observed that the vaccine-induced modulation of these markers resulted in the polarization of microglia into specific phenotypes different to the ones observed in the naive and adjuvant groups, where there is barely any correlation between markers implying a broad variety of microglia. Thus, the correlation of markers is due to the variant and its dose and not the effect of the adjuvant; low-dose α-syn, independently of variant (nitrated or not), induced a distinct type of brain microglia (CD54CD172aCD103+ CD200R-, 87 % of total), but when the dose of the α-syn was increased, this polarization of the microglia is affected by the variant : Nα-syn gave rise to a distinct phenotype by inducing the expression of CD4 on practically all brain microglia (97.32 %), while high-dose α-syn resulted in the loss of microglia polarization. Unfortunately, this CD4 expression was not detectable by immunohistochemistry, suggesting that its level of expression must be too low to be visible using this technique (i.e., how many CD4 molecules/microglia).

## Discussion

Great attention has been put into α-syn-based immunization therapies in PD, but little is known about the effect that α-syn, an autologous protein, has on the immune system. Indeed, we have previously shown that vaccination with low-dose α-syn and Freund’s adjuvant results in protection against α-syn-induced striatal pathology [3], but the mechanism behind this successful approach was unclear. Therefore, we have herein immunized naive Foxp3-RFP mice with human α-syn at two doses, low (15 μg, a dose equivalent to that used in rats in our previous study) or high (100 μg, to assess dose effect), with Freund’s adjuvant as before to determine the mechanism behind the successful vaccination approach. We have used mice instead of rats, as in our previous study, in order to benefit from the transgenic Foxp3-RFP strain and so easily follow Treg cells.

We examined the effect that α-syn had on the peripheral CD4 T cell pool and central nervous immune system (microglia) 5 days after the second immunization. Importantly, our study was undertaken in the absence of any human α-syn expression, pathology, or deposition in the brain.

### α-Synuclein modulates the naive/activated/memory frequencies of regulatory T cells

α-Syn immunization specifically changed the expression of CD127 and CCR6 in the Foxp3+ Treg cell population. Low-dose α-syn vaccination significantly increased the naive population (CD127-CCR6+) suggesting the generation of antigen-specific Tregs in the periphery; this expansion was reflected in the increase of total Foxp3+ cells. α-Syn at high dose had a tendency to induce memory Tregs, as it increased the fraction of CD127lo/negCCR6-Foxp3+ cells, and this correlated with an increase in CD103+ cells and increased phosphorylation of Stat5. IL-7R (CD127) is highly expressed by activated Tregs and correlates with the expression of CD103, ICOS, phospho-Stat5, and enhanced survival capacity [55]. In vitro, IL-7 signaling potentiates Treg function by increasing Foxp3, CD25, and CTLA-4 expression resulting in the down-regulation of its receptor CD127 [56]; these CD127lo memory Tregs are strongly suppressive [57]. Here, it is difficult to know whether the high-dose α-syn immunization induced the activation of the natural Treg pool or if newly generated α-syn-specific Tregs were activated due to the higher concentration of α-syn.

The peripheral immune system is used to sensing α-syn, which is constitutively secreted by enteric neurons in vitro [58], and it is naturally expressed in erythrocytes [59] and most immune cells [60]. With age, α-syn accumulates in healthy human peripheral blood mononuclear cells (PBMCs), an accumulation that is greater in PD patients, and compromises survival and function of these cells [61, 62]. Hence, the dose of α-syn, which the immune system encounters varies with age, in particular in PD patients, potentially affecting the way the immune system reacts to it. Indeed, it is known that a low antigen dose with suboptimal antigen presentation induces Tregs in the periphery and expands them [63]. Thus, our results suggest that low α-syn levels may expand the naturally occurring Tregs specific for the endogenous α-syn. This is supported by the non-significant increase in Foxp3 protein levels and the lack of antibodies against α-syn, suggesting a suboptimal TCR-mediated activation of CD4 T cells. At higher doses, α-syn may induce Treg activation (supported by the increased phospho-Stat5). When a pathological threshold of α-syn is attained, at which point the protein has likely been modified, tolerance towards α-syn may break, and a detrimental immune response that favors autoimmune inflammation (Th17) may be induced. Indeed, in previous work by Gendelman’s group, they immunized mice with 50 μg of nitrated α-syn, which resulted in the induction of Th17 T cells and defective Treg [40]. Thus, our previous vaccine approach proved beneficial because we increased tolerance towards α-syn through the expansion of naive Tregs. Our new results show that the dose employed is important, as increasing the dose results in a different immune response, and this might not prove protective. Together, the evidence suggests that in a pathological scenario such as PD, as brain pathology and the pathological state of α-syn progress, different Treg responses may be induced. These Tregs will have different capabilities to migrate to the brain, judging by their CCR6 expression. CCR6 is expressed by all Th17 T cells (autoimmune inflammation) and a subset of Tregs (effector/memory [64]), and in both cases, its expression is required to cross the blood-brain-barrier ([65], reviewed in [66, 67]).

### α-Synuclein-induced immune changes show association with dopamine signaling

It is well established that dopamine regulates the adaptive immune system (for a review see [11–13, 68, 69]). Interestingly, dopamine and α-syn can modify each other: interaction between oxidized dopamine and α-syn results in toxic compounds [36–39], which may putatively signal to the immune system. Furthermore, dopamine-modified α-syn does not induce TNF and nitric oxide by microglia, but instead has an anti-oxidant effect, something not seen with the oligomeric α-syn, further supporting the idea that dopamine and α-syn interaction can be meaningful to pathology [70]. In turn, α-syn oligomerization is promoted by dopamine, preventing its fibrillization (for a review see [17]). In healthy humans, it is estimated that there is 10 pg/ml of dopamine in serum, rising to 80 pg/ml during sickness, and such concentrations are able to alter T cell physiology [5]. DR signaling has been associated with particular T cell cytokine profiles depending on the specific dopamine receptor type involved, and particularly influences the Th17 vs. Treg balance [15, 16, 71–73]. We measured expression levels of DR-D3 and DR-D2, as DR-D3 signaling in CD4 T cells is associated with inflammation (IFNγ), while DR-D2 signaling with regulation of immune responses (IL-10) [69]. Interestingly, the absence of DR-D3 in CD4 T cells protects from MPTP-induced neuroinflammation [50], and DR-D2 knockout mice develop PD-like features [51]. This suggests that changes in dopamine levels during PD may crucially affect the peripheral immune system, in particular because these two receptors have very different affinities for dopamine, and thus if dopamine becomes scarce in PD, it will preferentially bind to DR-D3 (Ki ≈ 27 nM vs. 1705 nM for DR-D2, [74–76]). A recent paper showed that DR-D3 stimulation reduced cAMP levels and ERK2 phosphorylation consequently increasing CD4+ T cell activation and Th1-differentiation, respectively [77]. We observed here an increase in in DR-D3+ CD4 T cells in mice vaccinated with low-dose α-syn compared to naive in both the Treg and Th pool, again suggesting that low-dose α-syn boosts regulation of immune responses, and its signaling may have contributed to the expansion of naive Treg.

Surprisingly, the expression of CD103 and CCR6 was only observed in cells expressing DR-D2 and DR-D3, as effector cells negative for DRs were CCR6-CD103-, and these markers were expressed by only 10 % of the DR-D2/D3-negative Foxp3+ cells. This suggests a physiological function for both dopamine and α-syn in T cells, after all it is known that T cells express α-syn [35]. Of particular notice is the relation between DR-D2/D3 and CCR6 on Tregs because not only does it denote activation; it also allows Tregs to migrate. Indeed, low-dose α-syn reduced the expression of CCR6 on DR-expressing cells and induced CD103 specifically on DR-D3+ cells. This is a dose-specific effect, as at high α-syn dose, CD103 was upregulated in both DR populations. As mentioned above, CD103 is considered a marker for Treg memory; thus, our findings suggest that α-syn at low dose acts differently in DR-D2+ and DR-D3+ Tregs, promoting respectively activation/migration and memory induction, as well as increasing their capacity to mediate suppression through cell-cell contacts in both cases.

### Microglia respond to peripheral immune changes induced by α-synuclein

One of the remarkable findings of this study is that peripheral α-syn immunization modified brain microglia. This was dose-dependent and occurred despite the absence of any brain α-syn pathology, as a low dose of α-syn induced microglia polarization but not a high dose. This effect was variant-dependent, as Nα-syn had distinct effects on brain microglia. The phenomenon that microglia are affected by peripheral immune events, modulating how they handle ongoing neurodegenerative processes in brain, is well documented (for a review see [78, 79]). Our cytometric analysis included full brain microglia; however, our immunohistological data suggest that the microglia response is anatomically specific, with substantia nigra being particularly sensitive to changes in the peripheral immune system. This is particularly intriguing from a PD perspective since although α-syn pathology extends throughout the patient’s brain, it is mainly the dopaminergic neurons in substantia nigra that die, and this is also an important site of chronic microglia activation. The idea that microglia protein expression is anatomically heterogeneous, even under surveillance/homeostatic state, is not a novel concept [80, 81]. Some markers expressed by microglia are immunoregulatory, such as CD80/CD86, which gives co-stimulation to T cells, indicating that the brain is naturally equipped to interact with the peripheral immune system [82]. In PD patients, depending on the disease stage, different cytokines, T cells, and CD68 microglia have been observed in different brain regions [83]. This time-and-region specificity has also been seen in PD animal models [84, 85]. Whether this region specificity is related to higher α-syn levels in substantia nigra (reviewed in [86]), a difference in the neuronal ability to signal to the immune system, or to an intrinsic susceptibility of nigral microglia to peripheral immune changes is yet to be determined. However, our study suggests that α-syn in a low dose induces a specific type of microglia that co-expresses CD54 and CD172a, and at least half of this population is also CD4CD103+; thus, this type is distinct from the microglia observed under the other conditions tested. What this means for protection against α-synucleopathies is early to say, but it is well known that CD172a (SIRPα) regulates innate immune responses [87] and TNF production [88]; additionally, it has been involved in restricting neuroinflammation (reviewed in [89]). CD54 (ICAM-1) binds LFA-1 on T cells [90], so the generated microglia has the capacity to bind to T cells, but not to activate them, as the microglia generated do not express MHC II. This could therefore result in a situation where the generated microglia sequester T cells and prevent their activation. T cells will then neither be activated nor available for activation by an antigen-presenting cell. Lastly, CD103 expression by dendritic cells has been associated with the generation of Tregs [91, 92]. Thus, this type of microglia seems to have the potential to promote interactions with the adaptive immune system and hinder its activation, while also inhibiting innate immune processes, which is in concordance with the expansion of Treg cells in the periphery. These interactions may prove crucial for disease progression, as well as vaccination strategies; indeed, a study has recently been published showing lymphatic vessels surrounding the brain and draining directly to deep cervical lymph nodes [93].

An interesting observation is that although microglia expressing CD40L (CD154) were increased, it did not correlate with the other markers. CD40L gives microglia the putative ability to interact with B cells by binding CD40. Normally, CD40-CD40L induce B cell maturation, IgG production, and memory induction, but it also requires TCR binding of MHC II on B cells. This ability could be crucial to neuron survival, depending on whether CD40L+ microglia-B cell interactions result in the sequestering (due to lacking additional signals) or activation (induction of a humoral response in absence of T cells) of the B cells, as antigen deposition on dopaminergic neurons has been inversely correlated with neuronal survival [94].

### Can an α-synuclein-based immune therapy prove effective in Parkinson’s disease?

Our previous studies [3] and those from the group of Gendelman in the MPTP model [4, 95, 96] seem to indicate that correct priming of T cells, in particular the Treg compartment, may harness neuroinflammation and thus protect dopaminergic neurons. This theory has been further supported by the effects seen on adaptive immunity and microglia responses in the DR-D3 knockout MPTP mouse model, which further links detrimental CD4 T cell processes with dopamine signaling [50]. Indeed, animals lacking lymphocytes or CD4 T cells are partially protected against MPTP neurodegeneration [97]. Thus, we believe that a possible way to prevent/slow dopaminergic cell death is a vaccine therapy aimed to increase tolerance towards α-syn and modulate the microglia response. Why do we believe that such a therapy can prove beneficial when clinical trials using vaccination for Alzheimer disease were unsuccessful? We believe the immune processes happening in Alzheimer’s disease and in PD are putatively of very different nature, and what is protective (or detrimental) in one disease will not necessarily produce the same effect in the other. Whereas the strategy for Alzheimer’s disease has been to activate the immune system to react towards Aβ deposits, it is our hypothesis that a successful immunotherapy for PD will raise tolerance to α-syn, so that the brain can better handle the detrimental effects caused by α-syn malfunction/aggregation instead of initiating an auto-inflammatory response to eradicate the problem (i.e., malfunctioning neurons). Thus, the failure observed in the trials for Alzheimer’s disease vaccination does not necessarily herald defeat for PD. Furthermore, depletion of Tregs (i.e., breaking tolerance) is beneficial in a mouse model of Alzheimer’s, thus showing that the immune processes are indeed different in these neurodegenerative diseases [98].

## Conclusions

Our data shows distinctive immune responses upon the different immunization strategies. These responses were unique to the antigen (α-syn variants) and dose and were not mere standard immune responses to any type of antigen. Indeed, we have observed that a non-disease relevant peptide such lipopolysaccharide (LPS), a well-known proinflammogen, results in a significantly different response in the T cells (Olesen, Christiansen, Jensen, Otzen, Romero-Ramos and Sanchez-Guajardo, *Submitted*). This is in agreement with a previous study comparing the immune response after intracerebral injections of α-syn or LPS [99]. Furthermore, several groups have reported different immune responses in macrophages/microglia for the different type of α-syn (for a review see [60]).

We show herein that CD4 T cell profiles can be modulated by α-syn (with adjuvant) in a dose-dependent manner: α-Syn in a low dose expands the naive Treg population, thus suggesting that the mechanism behind our previously described successful vaccine approach was due to the induction of increased tolerance to α-syn. This immune modulation is dose-dependent, since increasing the dose of α-syn resulted in the expansion of the activated Treg pool. We have further shown that α-syn stimulation of T cells involves dopamine signaling in a yet undetermined manner regulating CD4 T cell homing and capacity to induce tolerance. Of particular interest is the relation of dopamine receptors and CCR6 expression, as all Th17 cells are CCR6+, and so are memory Tregs. Additionally, CCR6 signaling has been implicated in the conversion of Tregs into Foxp3+RORγt+ cells [100]. These events in the periphery affected microglia phenotype in the absence of brain α-syn pathology, also in a variant- and dose-dependent manner. Microglia modulation was neither due to CD4 T cell infiltration nor antigen deposition, and it was particularly different in substantia nigra as shown by morphological changes of immunostained microglia. We have thus shown that the immune system is sensitive to changes in α-syn and that antigen-specific immunoregulatory therapies based on modulating microglia responses by acting on the peripheral immune system are possible.

## Additional files

Additional file 1: Table S1. Antibodies. (PDF 158 kb) Additional file 2: Figure S1. CD11b immunohistochemistry. Representative striatum, cortex, and hippocampus photomicrographs stained for CD11b, where no apparent microgliosis was observed. ×10 magnification. Scale bar, 100 μm. (PDF 5.78 mb) Additional file 3: Figure S2. MHC II, CD4, and mouse IgG immunohistochemistry. A, B Representative photomicrographs of SN brain sections stained for MHC II. C–E Representative photomicrographs of brain sections stained for CD4. No CD4 staining corresponding to T cells was observed throughout the brain in any of the groups, however sporadic CD4+ microglia was found in the adjuvant (A) and α-syn low dose (B) groups. Additionally, sporadic CD4+ immunostaining associated to blood vessels was found in the α-syn high dose group (C). F&G Representative pictographs of brain sections stained for mouse IgG. Only in the α-syn high-dose group’s hippocampus was sporadic mouse IgG staining observed (F); however, blood vessels in all groups stained positive for IgG (G). A, C, D & F: ×20 magnification, scale bar in E, 10 μm. B, E, G: ×10 magnification, scale bar in D, 100 μm. (PDF 2.38 mb) Additional file 4: Figure S3. α-Synuclein immunohistochemistry. Representative substantia nigra photomicrographs stained for anti-human-α-synuclein. No staining was observed throughout the brain. ×10 magnification. Scale bar, 100 μm. (PDF 1.65 mb)

## Abbreviations

CSF: cerebrospinal fluid
DR: dopamine receptor
MPTP: 1-methyl-4-phenyl-1,2,3,6-tetrahydropyridine
Nα-syn: nitrated α-synuclein
PBMC: peripheral blood mononuclear cells
PD: Parkinson’s disease
RFP: red fluorescent protein
Th: CD4 effector T cell
Treg: regulatory T cell (CD4CD3Foxp3+)
α-syn: α-synuclein

## References

[1] Masliah E, Rockenstein E, Adame A, Alford M, Crews L, Hashimoto M (2005). Effects of alpha-synuclein immunization in a mouse model of Parkinson’s disease. Neuron.

[2] Masliah E, Rockenstein E, Mante M, Crews L, Spencer B, Adame A (2011). Passive immunization reduces behavioral and neuropathological deficits in an alpha-synuclein transgenic model of Lewy body disease. PLoS One.

[3] Sanchez-Guajardo V, Annibali A, Jensen PH, Romero-Ramos M (2013). Alpha-synuclein vaccination prevents the accumulation of Parkinson disease-like pathologic inclusions in striatum in association with regulatory T cell recruitment in a rat model. J Neuropathol Exp Neurol.

[4] Reynolds AD, Stone DK, Hutter JA, Benner EJ, Mosley RL, Gendelman HE (2010). Regulatory T cells attenuate Th17 cell-mediated nigrostriatal dopaminergic neurodegeneration in a model of Parkinson’s disease. J Immunol.

[5] Saha B, Mondal AC, Majumder J, Basu S, Dasgupta PS (2001). Physiological concentrations of dopamine inhibit the proliferation and cytotoxicity of human CD4+ and CD8+ T cells in vitro: a receptor-mediated mechanism. Neuroimmunomodulation.

[6] Nagai Y, Ueno S, Saeki Y, Soga F, Hirano M, Yanagihara T (1996). Decrease of the D3 dopamine receptor mRNA expression in lymphocytes from patients with Parkinson’s disease. Neurology.

[7] Levite M, Chowers Y, Ganor Y, Besser M, Hershkovits R, Cahalon L (2001). Dopamine interacts directly with its D3 and D2 receptors on normal human T cells, and activates beta1 integrin function. Eur J Immunol.

[8] Besser MJ, Ganor Y, Levite M (2005). Dopamine by itself activates either D2, D3 or D1/D5 dopaminergic receptors in normal human T-cells and triggers the selective secretion of either IL-10, TNFalpha or both. J Neuroimmunol.

[9] Ghosh MC, Mondal AC, Basu S, Banerjee S, Majumder J, Bhattacharya D (2003). Dopamine inhibits cytokine release and expression of tyrosine kinases, Lck and Fyn in activated T cells. Int Immunopharmacol.

[10] Sarkar C, Das S, Chakroborty D, Chowdhury UR, Basu B, Dasgupta PS (2006). Cutting edge: stimulation of dopamine D4 receptors induce T cell quiescence by up-regulating Kruppel-like factor-2 expression through inhibition of ERK1/ERK2 phosphorylation. J Immunol.

[11] Pacheco R, Prado CE, Barrientos MJ, Bernales S (2009). Role of dopamine in the physiology of T-cells and dendritic cells. J Neuroimmunol.

[12] Sarkar C, Basu B, Chakroborty D, Dasgupta PS, Basu S (2010). The immunoregulatory role of dopamine: an update. Brain Behav Immun.

[13] Romero-Ramos M, von Euler CM, Sanchez-Guajardo V (2014). Vaccination strategies for Parkinson disease: induction of a swift attack or raising tolerance?. Hum Vaccin Immunotherapeutics.

[14] Nakano K, Higashi T, Takagi R, Hashimoto K, Tanaka Y, Matsushita S (2009). Dopamine released by dendritic cells polarizes Th2 differentiation. Int Immunol.

[15] Prado C, Contreras F, Gonzalez H, Diaz P, Elgueta D, Barrientos M (2012). Stimulation of dopamine receptor D5 expressed on dendritic cells potentiates Th17-mediated immunity. J Immunol.

[16] Cosentino M, Fietta AM, Ferrari M, Rasini E, Bombelli R, Carcano E (2007). Human CD4+CD25+ regulatory T cells selectively express tyrosine hydroxylase and contain endogenous catecholamines subserving an autocrine/paracrine inhibitory functional loop. Blood.

[17] Leong SL, Cappai R, Barnham KJ, Pham CL (2009). Modulation of alpha-synuclein aggregation by dopamine: a review. Neurochem Res.

[18] Lee PH, Lee G, Park HJ, Bang OY, Joo IS, Huh K (2006). The plasma alpha-synuclein levels in patients with Parkinson’s disease and multiple system atrophy. J Neural Transm.

[19] Mollenhauer B (2014). Quantification of alpha-synuclein in cerebrospinal fluid: how ideal is this biomarker for Parkinson’s disease?. Parkinsonism Relat Disord.

[20] Desplats P, Lee HJ, Bae EJ, Patrick C, Rockenstein E, Crews L (2009). Inclusion formation and neuronal cell death through neuron-to-neuron transmission of alpha-synuclein. Proc Natl Acad Sci U S A.

[21] Lee SJ, Desplats P, Lee HJ, Spencer B, Masliah E (2012). Cell-to-cell transmission of alpha-synuclein aggregates. Methods Mol Biol.

[22] Luk KC, Kehm V, Carroll J, Zhang B, O’Brien P, Trojanowski JQ (2012). Pathological alpha-synuclein transmission initiates Parkinson-like neurodegeneration in nontransgenic mice. Science.

[23] Papachroni KK, Ninkina N, Papapanagiotou A, Hadjigeorgiou GM, Xiromerisiou G, Papadimitriou A (2007). Autoantibodies to alpha-synuclein in inherited Parkinson’s disease. J Neurochem.

[24] Maetzler W, Berg D, Synofzik M, Brockmann K, Godau J, Melms A (2011). Autoantibodies against amyloid and glial-derived antigens are increased in serum and cerebrospinal fluid of Lewy body-associated dementias. J Alzheimers Dis.

[25] Besong-Agbo D, Wolf E, Jessen F, Oechsner M, Hametner E, Poewe W (2013). Naturally occurring alpha-synuclein autoantibody levels are lower in patients with Parkinson disease. Neurology.

[26] Baba Y, Kuroiwa A, Uitti RJ, Wszolek ZK, Yamada T (2005). Alterations of T-lymphocyte populations in Parkinson disease. Parkinsonism Relat Disord.

[27] Bas J, Calopa M, Mestre M, Mollevi DG, Cutillas B, Ambrosio S (2001). Lymphocyte populations in Parkinson’s disease and in rat models of parkinsonism. J Neuroimmunol.

[28] Alberio T, Pippione AC, Zibetti M, Olgiati S, Cecconi D, Comi C (2012). Discovery and verification of panels of T-lymphocyte proteins as biomarkers of Parkinson’s disease. Scientific reports.

[29] Stevens CH, Rowe D, Morel-Kopp MC, Orr C, Russell T, Ranola M (2012). Reduced T helper and B lymphocytes in Parkinson’s disease. J Neuroimmunol.

[30] Gruden MA, Sewell RD, Yanamandra K, Davidova TV, Kucheryanu VG, Bocharov EV (2011). Immunoprotection against toxic biomarkers is retained during Parkinson’s disease progression. J Neuroimmunol.

[31] Prigione A, Begni B, Galbussera A, Beretta S, Brighina L, Garofalo R (2006). Oxidative stress in peripheral blood mononuclear cells from patients with Parkinson’s disease: negative correlation with levodopa dosage. Neurobiol Dis.

[32] Prigione A, Isaias IU, Galbussera A, Brighina L, Begni B, Andreoni S (2009). Increased oxidative stress in lymphocytes from untreated Parkinson’s disease patients. Parkinsonism Relat Disord.

[33] Battisti C, Formichi P, Radi E, Federico A (2008). Oxidative-stress-induced apoptosis in PBLs of two patients with Parkinson disease secondary to alpha-synuclein mutation. J Neurol Sci.

[34] Calopa M, Bas J, Callen A, Mestre M (2010). Apoptosis of peripheral blood lymphocytes in Parkinson patients. Neurobiol Dis.

[35] Shin EC, Cho SE, Lee DK, Hur MW, Paik SR, Park JH (2000). Expression patterns of alpha-synuclein in human hematopoietic cells and in Drosophila at different developmental stages. Molecules and cells.

[36] Nakaso K, Tajima N, Ito S, Teraoka M, Yamashita A, Horikoshi Y (2013). Dopamine-mediated oxidation of methionine 127 in alpha-synuclein causes cytotoxicity and oligomerization of alpha-synuclein. PLoS One.

[37] Lee HJ, Baek SM, Ho DH, Suk JE, Cho ED, Lee SJ (2011). Dopamine promotes formation and secretion of non-fibrillar alpha-synuclein oligomers. Exp Mol Med.

[38] Ha Y, Yang A, Lee S, Kim K, Liew H, Lee SH (2014). Dopamine and Cu+/2+ can induce oligomerization of alpha-synuclein in the absence of oxygen: two types of oligomerization mechanisms for alpha-synuclein and related cell toxicity studies. J Neurosci Res.

[39] Tavassoly O, Nokhrin S, Dmitriev OY, Lee JS (2014). Cu(II) and dopamine bind to alpha-synuclein and cause large conformational changes. The FEBS journal.

[40] Benner EJ, Banerjee R, Reynolds AD, Sherman S, Pisarev VM, Tsiperson V (2008). Nitrated alpha-synuclein immunity accelerates degeneration of nigral dopaminergic neurons. PLoS One.

[41] Reynolds AD, Stone DK, Mosley RL, Gendelman HE (2009). Nitrated {alpha}-synuclein-induced alterations in microglial immunity are regulated by CD4+ T cell subsets. J Immunol.

[42] Wan YY, Flavell RA (2005). Identifying Foxp3-expressing suppressor T cells with a bicistronic reporter. Proc Natl Acad Sci U S A.

[43] Nielsen SB, Macchi F, Raccosta S, Langkilde AE, Giehm L, Kyrsting A (2013). Wildtype and A30P mutant alpha-synuclein form different fibril structures. PLoS One.

[44] Smith EL, Finney HM, Nesbitt AM, Ramsdell F, Robinson MK (2006). Splice variants of human FOXP3 are functional inhibitors of human CD4+ T-cell activation. Immunology.

[45] Armentero MT, Levandis G, Nappi G, Bazzini E, Blandini F (2006). Peripheral inflammation and neuroprotection: systemic pretreatment with complete Freund’s adjuvant reduces 6-hydroxydopamine toxicity in a rodent model of Parkinson’s disease. Neurobiol Dis.

[46] Yong J, Lacan G, Dang H, Hsieh T, Middleton B, Wasserfall C (2011). BCG vaccine-induced neuroprotection in a mouse model of Parkinson’s disease. PLoS One.

[47] Outeiro TF, Klucken J, Bercury K, Tetzlaff J, Putcha P, Oliveira LM (2009). Dopamine-induced conformational changes in alpha-synuclein. PLoS One.

[48] Cappai R, Leck SL, Tew DJ, Williamson NA, Smith DP, Galatis D (2005). Dopamine promotes alpha-synuclein aggregation into SDS-resistant soluble oligomers via a distinct folding pathway. FASEB J.

[49] Conway KA, Rochet JC, Bieganski RM, Lansbury PT (2001). Kinetic stabilization of the alpha-synuclein protofibril by a dopamine-alpha-synuclein adduct. Science.

[50] Gonzalez H, Contreras F, Prado C, Elgueta D, Franz D, Bernales S (2013). Dopamine receptor D3 expressed on CD4+ T cells favors neurodegeneration of dopaminergic neurons during Parkinson’s disease. J Immunol.

[51] Tinsley RB, Bye CR, Parish CL, Tziotis-Vais A, George S, Culvenor JG (2009). Dopamine D2 receptor knockout mice develop features of Parkinson disease. Ann Neurol.

[52] Reboldi A, Coisne C, Baumjohann D, Benvenuto F, Bottinelli D, Lira S (2009). C-C chemokine receptor 6-regulated entry of TH-17 cells into the CNS through the choroid plexus is required for the initiation of EAE. Nat Immunol.

[53] Allakhverdi Z, Fitzpatrick D, Boisvert A, Baba N, Bouguermouh S, Sarfati M (2006). Expression of CD103 identifies human regulatory T-cell subsets. J Allergy Clin Immunol.

[54] Huang G, Wang Y, Chi H (2013). Control of T cell fates and immune tolerance by p38alpha signaling in mucosal CD103+ dendritic cells. J Immunol.

[55] Simonetta F, Chiali A, Cordier C, Urrutia A, Girault I, Bloquet S (2010). Increased CD127 expression on activated FOXP3+CD4+ regulatory T cells. Eur J Immunol.

[56] Di Caro V, D’Anneo A, Phillips B, Engman C, Harnaha J, Lakomy R (2011). Interleukin-7 matures suppressive CD127(+) forkhead box P3 (FoxP3)(+) T cells into CD127(-) CD25(high) FoxP3(+) regulatory T cells. Clin Exp Immunol.

[57] Beyer M, Classen S, Endl E, Kochanek M, Weihrauch MR, Debey-Pascher S (2011). Comparative approach to define increased regulatory T cells in different cancer subtypes by combined assessment of CD127 and FOXP3. Clin Dev Immunol.

[58] Paillusson S, Clairembault T, Biraud M, Neunlist M, Derkinderen P (2013). Activity-dependent secretion of alpha-synuclein by enteric neurons. J Neurochem.

[59] Barbour R, Kling K, Anderson JP, Banducci K, Cole T, Diep L (2008). Red blood cells are the major source of alpha-synuclein in blood. Neurodegener Dis.

[60] Sanchez-Guajardo V, Barnum CJ, Tansey MG, Romero-Ramos M (2013). Neuroimmunological processes in Parkinson’s disease and their relation to alpha-synuclein: microglia as the referee between neuronal processes and peripheral immunity. ASN neuro.

[61] Kim S, Jeon BS, Heo C, Im PS, Ahn TB, Seo JH (2004). Alpha-synuclein induces apoptosis by altered expression in human peripheral lymphocyte in Parkinson’s disease. FASEB J.

[62] Gardai SJ, Mao W, Schule B, Babcock M, Schoebel S, Lorenzana C (2013). Elevated alpha-synuclein impairs innate immune cell function and provides a potential peripheral biomarker for Parkinson’s disease. PLoS One.

[63] Kretschmer K, Apostolou I, Hawiger D, Khazaie K, Nussenzweig MC, von Boehmer H (2005). Inducing and expanding regulatory T cell populations by foreign antigen. Nat Immunol.

[64] Kleinewietfeld M, Puentes F, Borsellino G, Battistini L, Rotzschke O, Falk K (2005). CCR6 expression defines regulatory effector/memory-like cells within the CD25(+)CD4+ T-cell subset. Blood.

[65] Yamazaki T, Yang XO, Chung Y, Fukunaga A, Nurieva R, Pappu B (2008). CCR6 regulates the migration of inflammatory and regulatory T cells. J Immunol.

[66] Comerford I, Bunting M, Fenix K, Haylock-Jacobs S, Litchfield W, Harata-Lee Y (2010). An immune paradox: how can the same chemokine axis regulate both immune tolerance and activation?: CCR6/CCL20: a chemokine axis balancing immunological tolerance and inflammation in autoimmune disease. Bioessays.

[67] Hedrick MN, Lonsdorf AS, Hwang ST, Farber JM (2010). CCR6 as a possible therapeutic target in psoriasis. Expert Opin Ther Targets.

[68] Pacheco R, Riquelme E, Kalergis AM (2010). Emerging evidence for the role of neurotransmitters in the modulation of T cell responses to cognate ligands. Cent Nerv Syst Agents Med Chem.

[69] Levite M (2008). Neurotransmitters activate T-cells and elicit crucial functions via neurotransmitter receptors. Curr Opin Pharmacol.

[70] Beraud D, Hathaway HA, Trecki J, Chasovskikh S, Johnson DA, Johnson JA (2013). Microglial activation and antioxidant responses induced by the Parkinson’s disease protein alpha-synuclein. J Neuroimmune Pharmacol.

[71] Ferreira TB, Kasahara TM, Barros PO, Vieira MM, Bittencourt VC, Hygino J (2011). Dopamine up-regulates Th17 phenotype from individuals with generalized anxiety disorder. J Neuroimmunol.

[72] Kipnis J, Cardon M, Avidan H, Lewitus GM, Mordechay S, Rolls A (2004). Dopamine, through the extracellular signal-regulated kinase pathway, downregulates CD4+CD25+ regulatory T-cell activity: implications for neurodegeneration. J Neurosci.

[73] Nakano K, Higashi T, Hashimoto K, Takagi R, Tanaka Y, Matsushita S (2008). Antagonizing dopamine D1-like receptor inhibits Th17 cell differentiation: preventive and therapeutic effects on experimental autoimmune encephalomyelitis. Biochem Biophys Res Commun.

[74] Strange PG (2001). Antipsychotic drugs: importance of dopamine receptors for mechanisms of therapeutic actions and side effects. Pharmacol Rev.

[75] Wu J, Hablitz JJ (2005). Cooperative activation of D1 and D2 dopamine receptors enhances a hyperpolarization-activated inward current in layer I interneurons. J Neurosci.

[76] Malmberg A, Jackson DM, Eriksson A, Mohell N (1993). Unique binding characteristics of antipsychotic agents interacting with human dopamine D2A, D2B, and D3 receptors. Mol Pharmacol.

[77] Franz D, Contreras F, Gonzalez H, Prado C, Elgueta D, Figueroa C (2015). Dopamine receptors D3 and D5 regulate CD4(+)T-cell activation and differentiation by modulating ERK activation and cAMP production. J Neuroimmunol.

[78] Perry VH, Nicoll JA, Holmes C (2010). Microglia in neurodegenerative disease. Nat Rev Neurol.

[79] Doorn KJ, Lucassen PJ, Boddeke HW, Prins M, Berendse HW, Drukarch B (2012). Emerging roles of microglial activation and non-motor symptoms in Parkinson’s disease. Prog Neurobiol.

[80] Hanisch UK (2013). Functional diversity of microglia—how heterogeneous are they to begin with?. Front Cell Neurosci.

[81] Hanisch UK, Kettenmann H (2007). Microglia: active sensor and versatile effector cells in the normal and pathologic brain. Nat Neurosci.

[82] de Haas AH, Boddeke HW, Biber K (2008). Region-specific expression of immunoregulatory proteins on microglia in the healthy CNS. Glia.

[83] Garcia-Esparcia P, Llorens F, Carmona M, Ferrer I (2014). Complex deregulation and expression of cytokines and mediators of the immune response in Parkinson’s disease brain is region dependent. Brain Pathol.

[84] Mitra S, Chakrabarti N, Bhattacharyya A (2011). Differential regional expression patterns of alpha-synuclein, TNF-alpha, and IL-1beta; and variable status of dopaminergic neurotoxicity in mouse brain after Paraquat treatment. J Neuroinflammation.

[85] Watson MB, Richter F, Lee SK, Gabby L, Wu J, Masliah E (2012). Regionally-specific microglial activation in young mice over-expressing human wildtype alpha-synuclein. Exp Neurol.

[86] Ulusoy A, Di Monte DA (2013). alpha-Synuclein elevation in human neurodegenerative diseases: experimental, pathogenetic, and therapeutic implications. Mol Neurobiol.

[87] Barclay AN, Brown MH (2006). The SIRP family of receptors and immune regulation. Nat Rev Immunol.

[88] Smith RE, Patel V, Seatter SD, Deehan MR, Brown MH, Brooke GP (2003). A novel MyD-1 (SIRP-1alpha) signaling pathway that inhibits LPS-induced TNFalpha production by monocytes. Blood.

[89] Zhang H, Li F, Yang Y, Chen J, Hu X (2015). SIRP/CD47 signaling in neurological disorders. Brain Res.

[90] Van Seventer GA, Shimizu Y, Horgan KJ, Shaw S (1990). The LFA-1 ligand ICAM-1 provides an important costimulatory signal for T cell receptor-mediated activation of resting T cells. J Immunol.

[91] Flores-Langarica A, Marshall JL, Hitchcock J, Cook C, Jobanputra J, Bobat S (2012). Systemic flagellin immunization stimulates mucosal CD103+ dendritic cells and drives Foxp3+ regulatory T cell and IgA responses in the mesenteric lymph node. J Immunol.

[92] Annacker O, Coombes JL, Malmstrom V, Uhlig HH, Bourne T, Johansson-Lindbom B (2005). Essential role for CD103 in the T cell-mediated regulation of experimental colitis. J Exp Med.

[93] Louveau A, Smirnov I, Keyes TJ, Eccles JD, Rouhani SJ, Peske JD et al. Structural and functional features of central nervous system lymphatic vessels. Nature. 2015. doi:10.1038/nature14432. 10.1038/nature14432 PMC4506234 26030524

[94] Orr CF, Rowe DB, Mizuno Y, Mori H, Halliday GM (2005). A possible role for humoral immunity in the pathogenesis of Parkinson’s disease. Brain.

[95] Reynolds AD, Banerjee R, Liu J, Gendelman HE, Mosley RL (2007). Neuroprotective activities of CD4+CD25+ regulatory T cells in an animal model of Parkinson’s disease. J Leukoc Biol.

[96] Laurie C, Reynolds A, Coskun O, Bowman E, Gendelman HE, Mosley RL (2007). CD4+ T cells from copolymer-1 immunized mice protect dopaminergic neurons in the 1-methyl-4-phenyl-1,2,3,6-tetrahydropyridine model of Parkinson’s disease. J Neuroimmunol.

[97] Brochard V, Combadiere B, Prigent A, Laouar Y, Perrin A, Beray-Berthat V (2009). Infiltration of CD4+ lymphocytes into the brain contributes to neurodegeneration in a mouse model of Parkinson disease. J Clin Invest.

[98] Baruch K, Rosenzweig N, Kertser A, Deczkowska A, Sharif AM, Spinrad A (2015). Breaking immune tolerance by targeting Foxp3(+) regulatory T cells mitigates Alzheimer’s disease pathology. Nat Commun.

[99] Couch Y, Alvarez-Erviti L, Sibson NR, Wood MJ, Anthony DC (2011). The acute inflammatory response to intranigral alpha-synuclein differs significantly from intranigral lipopolysaccharide and is exacerbated by peripheral inflammation. J Neuroinflammation.

[100] Lochner M, Peduto L, Cherrier M, Sawa S, Langa F, Varona R (2008). In vivo equilibrium of proinflammatory IL-17+ and regulatory IL-10+ Foxp3+ RORgamma t + T cells. J Exp Med.

