# Supplementary material for: α-Synuclein vaccination modulates regulatory T cell activation and microglia in the absence of brain pathology
Source: J Neuroinflammation. 2016 Apr 7;13:74. doi: 10.1186/s12974-016-0532-8 (PMC4825077; doi:10.1186/s12974-016-0532-8)
Supplement: Additional file 1: Table S1. — Antibodies. (PDF 158 kb) [file 12974_2016_532_MOESM1_ESM.pdf]

Additional file 1: Table S1. Antibodies

Antibody	*Coupled to*	*Dilution*	*Made in*	*Clone*	*Manufacturer*	*Function*
**Flow cytometry**						
CD4	AF700	1:800	Rat	RM4-5	BD Pharmingen	TCR complex, binds MHCII
CD3ε	HV500	1:100	Syrian Hamster	500A2	BD Horizon	TCR complex, kinase
CD25	PE-Cy7	1:200	Rat	PC61	BD Pharmingen	IL-2Rα, T cell activation / Treg survival
DR-D2	Purified	1:50	Goat	Polyclonal	Abcam	Dopamine receptor D2
DR-D3	Purified	1:50	Rabbit	Polyclonal	Abcam	Dopamine receptor D3
CD127	Biotin	1:100	Rat	A7R34	eBioscience	IL-7Rα, effector/memory T cell
CD196 (CCR6)	BV421	1:50	Armenian Hamster	29-2L17	Biolegend	CCR6 Chemokine receptor. Migration & homeostasis of lymphocytes.
CD103	PerCP-Cy5.5	1:100	Armenian Hamster	2E7	BioLegend	Lectin, tolerance. Treg express high levels. Th cells use it to attach to endothelium and extravasate
CD11b	PE-Cy7	1:800	Rat	M1/70	BD Pharmingen	Lectin, binds ICAM-1. When in complex with CD18, forms CR3 (binds C3bi).<br>Macrophage/monocyte cells
CD11c	AF700	1:800	Armenian Hamster	N418	BioLegend	Lectin, When in complex with CD18, forms CR3 (binds C3bi).<br>Dendritic & NK cells
CD54 (ICAM-1)	APC	1:100	Hamster	3E2	BD Pharmingen	ICAM-1, binds CD11b/CD18 & CD11c/CD18, involved in MHC-independent responses to weak immunogenic tumor cells
CD154 (CD40L)	Biotin	1:50	Armenian Hamster	MR1	BD Pharmingen	CD40 ligand, binds CD40 on B cells for T cell induction of memory T cells
CD200R	PE	1:200	Rat	OX-110	Biolegend	Ox-2R, Suppresses inflammatory reactions on macrophages, Neuron-glia inhibitory interaction

MHC II (I-A/I-E)	eFluo450	1:100	Rat	M5/114.15.2	eBioscience	Antigen presentation, binds CD4/TCR
CD172a	FITC	1:100	Rat	P84	BD Pharmingen	Adhesion molecule. Neurons, retina macrophages. Regulates phagocytosis and synaptic activity. Microglia-neuron regulatory interactions.
CD4	APC-H7	1:200	Rat	GK1.5	BD Pharmingen	TCR complex, binds MHCII
Streptavidin	QDot605	1:200				Secondary antibody
Anti-rabbit IgG	AF647	1:100	Donkey	n.a.	Abcam	Secondary antibody
Anti-goat IgG	FITC	1:50	Donkey	n.a.	Abcam	Secondary antibody
CD16/CD32	Purified	1:50	Rat	2.4G2	BD Pharmingen	Fc block, binds to receptors for IgG
**Western blot**						
Foxp3	Purified	1:50	Mouse	150D	BioLegend	Transcription factor specific for Treg
Foxp3	Purified	1:300	Rabbit	n.s.m.	Aviva Systems Biology	Transcription factor specific for Treg
RORγt	Purified	1:100	Mouse		BD Transduction	Transcription factor specific for Th17 cells
Stat3	Purified	1:3000	Mouse	84/Stat3	BD Transduction	Transcription factor
Stat3-pS727	Purified	1:500	Mouse	49/p-Stat3	BD Transduction	Phosphorylation due to EGF & IL-6, Binds c-fos promoter
Stat5	Purified	1:500	Mouse	89/Stat5	BD Transduction	Transcription factor
Stat5-pY694	Purified	1:50	Mouse	47	BD Transduction	Involved in IL-2-induced activation of T cells
β-actin	Purified	1:20000	Mouse	AC-15	Sigma-Aldrich	
Anti-rabbit IgG	HRP	1:2000	Swine	polyclonal	Dako Cytomation	Secondary antibody
Anti-mouse IgG	HRP	1:2000	Rabbit	polyclonal	DakoCytomation	Secondary antibody
**Immunohistochemistry**						
a-syn	Purified	1:4000	Rabbit	n.s.m.	Abcam	Residues 118-123

(Ab138501)						
CD11b	Purified	1:200	Rat	M1/70.15	AbD Serotec	See Flow Cytometry
CD4	Purified	1:200	Rat	GK1.5	AbD Serotec	See Flow Cytometry
MHC II						See Flow Cytometry
Anti-mouse IgG	Biotin	1:2000	Horse	Polyclonal	Vector Laboratories	Recognized antibodies of mouse origin
Anti-rat IgG	Biotin	1:200	Goat	Polyclonal	Vector Laboratories	Secondary antibody
Anti-rabbit IgG	Biotin	1:200	Donkey	Polyclonal	Vector Laboratories	Secondary antibody
**Anti-a-syn ELISA**						
human-a-syn	Purified	1:1000-128.000	Mouse	4B12	Covance	For standard curve
Anti-mouse IgG	HRP	1:2000	Rabbit	Polyclonal	DAKO	

n.s.m. Not supplied by the manufacturer; n.a. Not applicable.

BD Bioscience, Belgium; Dako Cytomation, Glostryp, Denmark; Aviva Biology Systems, San Diego, CA, USA; BioLegend, San Diego, CA, USA; eBioscience, San Diego, CA, USA; Abcam, Cambridge, UK; Vector Laboratories, Burlingame, CA, USA; AbD Serotec, a division of MorphoSys, Munich, Germany; Sigma-Aldrich, St. Louis, MO, USA; Pierce Antibodies, Rockford, IL, USA
