# Supplementary figures and images for: α-Synuclein vaccination modulates regulatory T cell activation and microglia in the absence of brain pathology
Source: J Neuroinflammation. 2016 Apr 7;13:74. doi: 10.1186/s12974-016-0532-8 (PMC4825077; doi:10.1186/s12974-016-0532-8)

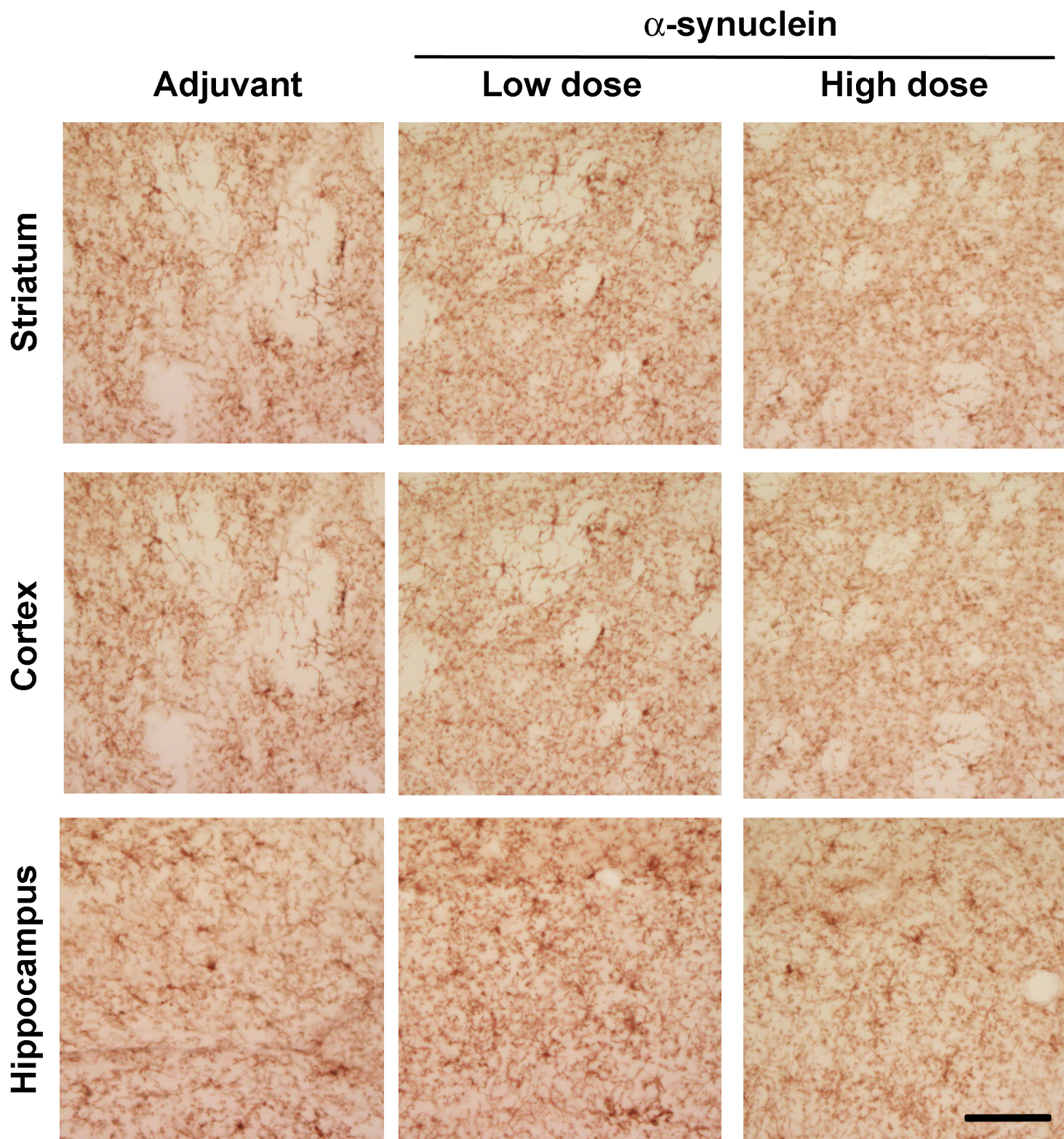

Supp.Fig.1

Supplement: Additional file 2: Figure S1. — CD11b immunohistochemistry. Representative striatum, cortex, and hippocampus photomicrographs stained for CD11b, where no apparent microgliosis was observed. ×10 magnification. Scale bar, 100 μm. (PDF 5.78 mb) [file 12974_2016_532_MOESM2_ESM.pdf]

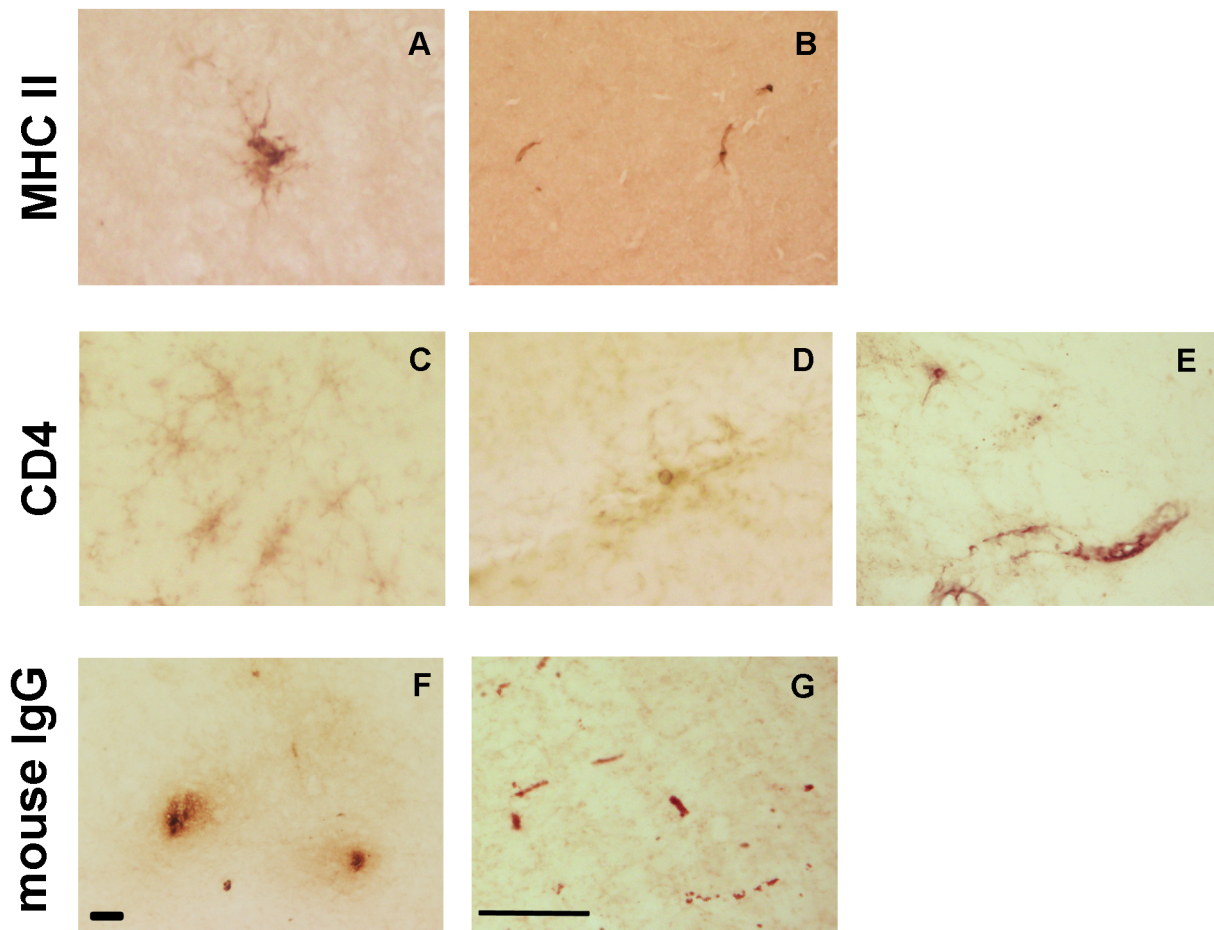

**Supp.Fig.2**

Supplement: Additional file 3: Figure S2. — MHC II, CD4, and mouse IgG immunohistochemistry. A, B Representative photomicrographs of SN brain sections stained for MHC II. C–E Representative photomicrographs of brain sections stained for CD4. No CD4 staining corresponding to T cells was observed throughout the brain in any of the groups, however sporadic CD4+ microglia was found in the adjuvant (A) and α-syn low dose (B) groups. Additionally, sporadic CD4+ immunostaining associated to blood vessels was found in the α-syn high dose group (C). F&G Representative pictographs of brain sections stained for mouse IgG. Only in the α-syn high-dose group’s hippocampus was sporadic mouse IgG staining observed (F); however, blood vessels in all groups stained positive for IgG (G). A, C, D & F: ×20 magnification, scale bar in E, 10 μm. B, E, G: ×10 magnification, scale bar in D, 100 μm. (PDF 2.38 mb) [file 12974_2016_532_MOESM3_ESM.pdf]

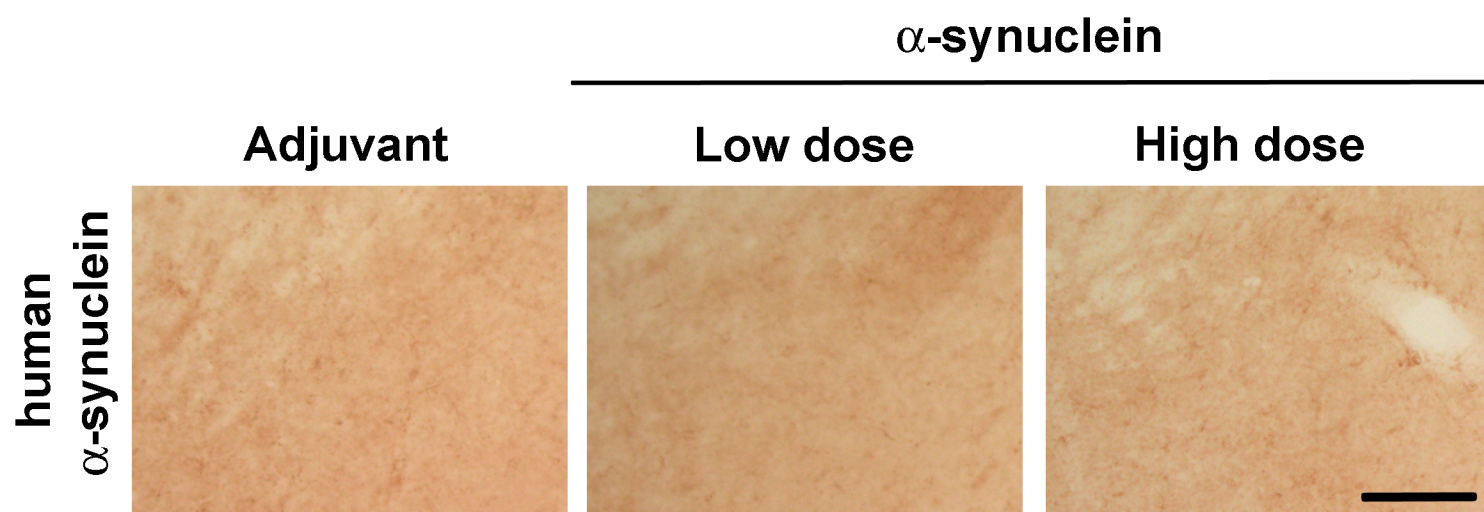

Supp.Fig.3

Supplement: Additional file 4: Figure S3. — α-Synuclein immunohistochemistry. Representative substantia nigra photomicrographs stained for anti-human-α-synuclein. No staining was observed throughout the brain. ×10 magnification. Scale bar, 100 μm. (PDF 1.65 mb) [file 12974_2016_532_MOESM4_ESM.pdf]
